# Multiresidue method for the determination of critically and highly important classes of antibiotics and their metabolites in agricultural soils and sewage sludge

**DOI:** 10.1007/s00216-023-04982-3

**Published:** 2023-10-17

**Authors:** Carmen Mejías, Juan Luis Santos, Julia Martín, Irene Aparicio, Esteban Alonso

**Affiliations:** Departamento de Química Analítica, Escuela Politécnica Superior, Universidad de Sevilla, C/ Virgen de África, 7, 41011 Seville, Spain

**Keywords:** Antibiotics, Metabolites, Soil, Sewage sludge, Compost, LC–MS/MS

## Abstract

In this paper, a method is proposed for the determination of antibiotics classified by the World Health Organization as critically important (four macrolides and three quinolones) and highly important (one tetracycline, one diaminopyridine, and three sulfonamides) and eight of their metabolites. The method is based on ultrasound-assisted extraction, dispersive solid-phase extraction clean-up, and analytical determination by liquid chromatography–tandem mass spectrometry. Variables affecting each stage of the analytical method were thoroughly optimised. The method was validated for its application to sewage sludge from different treatment stages (non-treated sludge: primary and secondary sludge; and treated sludge: digested sludge and compost) and to agricultural soil. Limits of quantification were in the range of 0.03–7.50 ng g^−1^ dry weight (dw) for most of the compounds. Accuracy values were in the range of 70–102%. Precision was below 17%. The application of the method to real samples revealed that macrolides and fluoroquinolones were the antibiotic classes at the highest concentrations in all types of samples. The lowest concentrations of antibiotics were measured in compost (highest concentration: 27 ng g^−1^ dw, corresponding to norfloxacin) and soil samples (highest concentration: 93 ng g^−1^ dw, corresponding to a metabolite of clarithromycin). The proposed method is the first developed to date for the determination of multiclass antibiotics and their main metabolites in sludge from different treatment stages. The method can provide a useful tool for obtaining information about antibiotics in sewage sludge prior to its application to agricultural soils and in agricultural soils.

**Graphical Abstract:**

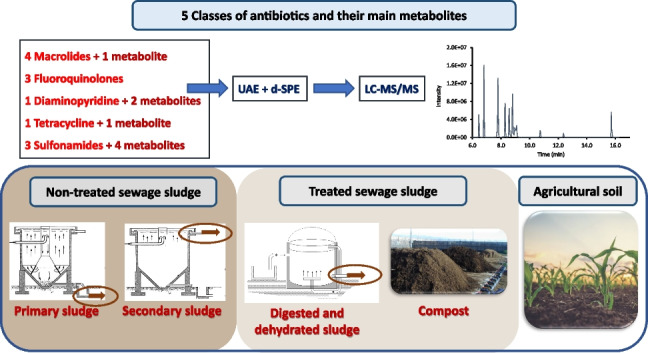

**Supplementary Information:**

The online version contains supplementary material available at 10.1007/s00216-023-04982-3.

## Introduction

Antibiotics are a group of pharmaceutical compounds widely used to prevent and treat bacterial infections in human and veterinary medicine. After their administration, they are excreted through the urine and/or faeces as parent compounds and/or metabolites. Because of their extended use—and often overuse and misuse—parent compounds and their metabolites are continuously discharged into the environment mainly through effluents from wastewater treatment plants (WWTP). They can also be accumulated onto sewage sludge generated during wastewater treatments [1–3]. This fact is of special concern when sewage sludge is applied onto agricultural soils as fertiliser [4]. Antibiotics can affect (i) microorganisms responsible for biological wastewater treatments; (ii) microorganisms in sewage sludge-amended soils; (iii) crops grown in soils irrigated with treated wastewater or fertiliser with sewage sludge; (iv) and even human health if they are taken up by edible crops. In addition, the World Health Organization warned in 2020 that antibiotic resistance is rising to such dangerously high levels that a growing list of infections are becoming harder and sometimes impossible to treat [5].

The application of sewage sludge onto agricultural soils is a common practice in many countries due to its valuable agronomic properties. For instance, more than 50% of treated sludge annually generated in Bulgaria, Ireland, Spain, the United Kingdom, Norway and Albania during 2012–2015 was applied as fertiliser onto agricultural soils [6]. According to the Environmental Protection Agency, 43% of generated sludge was applied onto agricultural and non-agricultural soils in the United States in 2021 [7]. Therefore, the antibiotic content of sewage sludge and agricultural soils should be monitored not only to prevent direct environmental risks and human health effects [1, 8, 9], but also to prevent the development of antibiotic-resistant bacteria (ARB) and antibiotic resistance genes (ARGs) in soils [8, 10, 11].

To date, most of the analytical methods reported for the determination of antibiotics in sewage sludge and soils have been developed for just one class of antibiotics [12–15] and type of sample (soil [16–18] or sludge [19, 20]). In addition, the few methods including the determination of antibiotic metabolites are mainly focused on sulfonamide metabolites [15, 16, 21]. Extraction methods reported for the determination of antibiotic residues in sludge and soils are based on ultrasound-assisted extraction (UAE) [16], microwave assisted extraction (MAE) [20], pressurised liquid extraction (PLE) [21] and QuEChERS (quick, easy, cheap, effective, rugged and safe) [22, 23] and, to a lesser extent, on matrix solid-phase dispersion (MSPD) [14, 24] and vortex-assisted solid–liquid extraction [18]. Extracts are commonly subjected to clean-up by solid-phase extraction (SPE) and are analysed by liquid chromatography–tandem mass spectrometry (LC–MS/MS) [1].

The aim of this work was to develop an analytical method suitable for application in routine control of multiclass high-concern antibiotics and their main metabolites in agricultural soils and in sludge. The antibiotics selected included four macrolides [azithromycin (AZM), clarithromycin (CLM), erythromycin (ERY) and roxithromycin (RXM)], three fluoroquinolones [ciprofloxacin (CIP), enrofloxacin (ENR) and norfloxacin (NOR)], a tetracycline [tetracycline (TC)], a diaminopyridine [trimethoprim (TMP)] and three sulfonamides [sulfadiazine (SDZ), sulfamethazine (SMZ) and sulfamethoxazole (SMX)]. Selected compounds belong to antibiotic classes categorised by the World Health Organization as critically important (macrolides and quinolones) or highly important (tetracyclines, diaminopyridine, and sulfonamides) [25] and are among the antibiotics most frequently detected in WWTPs [26]. In addition, many of them were included in the second (AZM, ERY, CLM and CIP) [27] and third (SMX and TMP) [28] European Union Watch Lists of contaminants of emerging concern in the aquatic environment. The method involves low-cost and easy-to-perform sample treatment techniques (UAE and d-SPE clean-up) and analytical determination by LC–MS/MS. Extraction and clean-up techniques were selected because they do not require expensive equipment, as PLE, MAE and SPE do; they are easy to perform and are commonly used in routine control laboratories; they do not generate plastic waste as the MSPD and SPE techniques do; and they allow the simultaneous treatment of many samples. The method was validated for its application to sludge from different treatment stages (non-treated sludge: primary and secondary sludge; treated sludge: digested sludge and compost) and to agricultural soils. To our knowledge, this is the first analytical method for multiclass determination of high environmental and health concern antibiotics and their metabolites in agricultural soils, treated sludge and non-treated sludge.

## Materials and methods

### Chemicals and reagents

High-purity standards of *N*^4^-acetylsulfadiazine (AcSDZ, > 99.0%), *N*^4^-acetylsulfamethoxazole (AcSMX, ≥ 98.5%), CIP (≥ 98.0%), ENR (≥ 98.5%), ROX (≥ 98.0%), SDZ (≥ 99.0%), SMZ (≥ 99.0%) and TC (≥ 95.0%) were provided by Sigma-Aldrich (Steinheim, Germany). *N*^4^-Acetylsulfamethazine (AcSMZ, ≥ 98.0%), sulfamethoxazole *N*^4^-glucoside (SMX-GL, > 99.0%), *N*-desmethylclarithromycin (DM-CLM, ≥ 96.0%), 3-desmethyltrimethoprim (DM-TMP, ≥ 98.0%) and 4-hydroxytrimethoprim (4-OH-TMP, ≥ 97.0%) were purchased from Toronto Research Chemicals (Toronto, Canada). CLM (≥ 98.0%) and ERY (≥ 98.0%) were supplied by Tokyo Chemicals Industry (Fukaya, Saitama). NOR (≥ 99.1%), SMX (≥ 99.5%) and TMP (≥ 99.5%) were provided by Dr Ehrenstorfer GmbH (Augsburg, Germany). 4-Epitetracycline (EP-TC, > 99.0%) was purchased from the WHO Centre for Chemical Reference Substances (Strasbourg, France). AZM (> 99.0%) was provided by the European Pharmacopoeia Reference Standard (Strasbourg, France). Chemical structures and physical–chemical properties of the target compounds can be seen in Table 1. Isotopically marked compounds [erythromycin-(*N*,*N*-dimethyl-^13^C_2_) (ERY-^13^C, ≥ 99.0%), ofloxacin-d_3_ (OFL-d_3_, ≥ 99.0%), sulfamethoxazole-(phenyl-^13^C_6_) (SMX-^13^C, ≥ 99.0%)], used as surrogate standards, were supplied by Sigma-Aldrich (Steinheim, Germany). Florisil^®^ and ammonium formate were provided by Sigma-Aldrich (Steinheim, Germany). Primary-secondary amine (PSA) and C18 were supplied by Scharlab (Barcelona, Spain). Formic acid (> 98.0%) was purchased from Panreac (Barcelona, Spain). All the reagents were of high purity and analytical grade. LC–MS-grade acetonitrile, methanol (MeOH), acetone and water were supplied by Merck (Darmstadt, Germany).

**Table 1:**
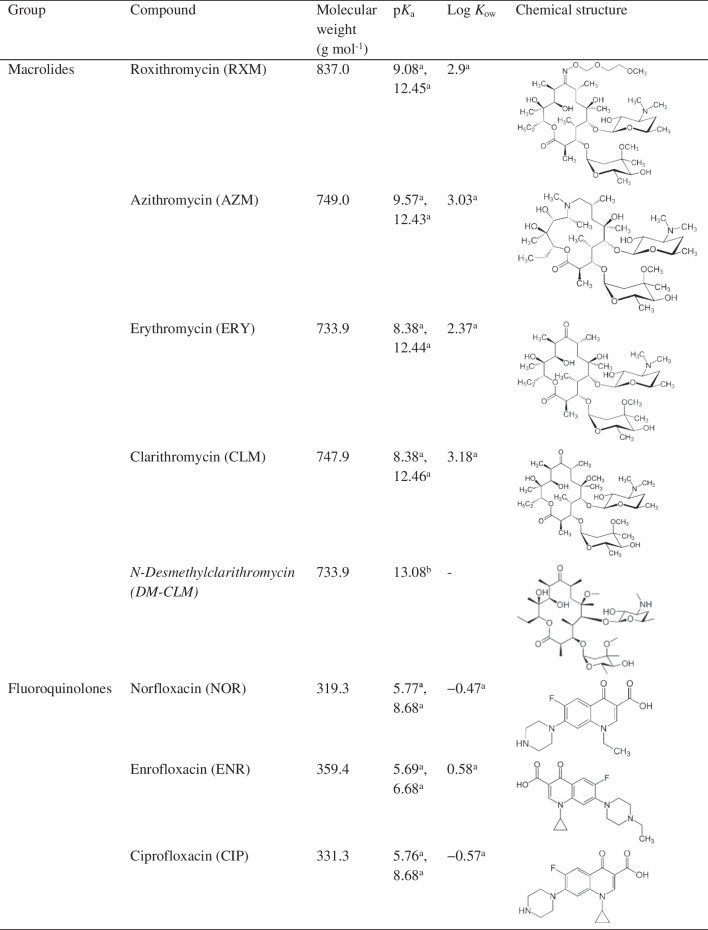

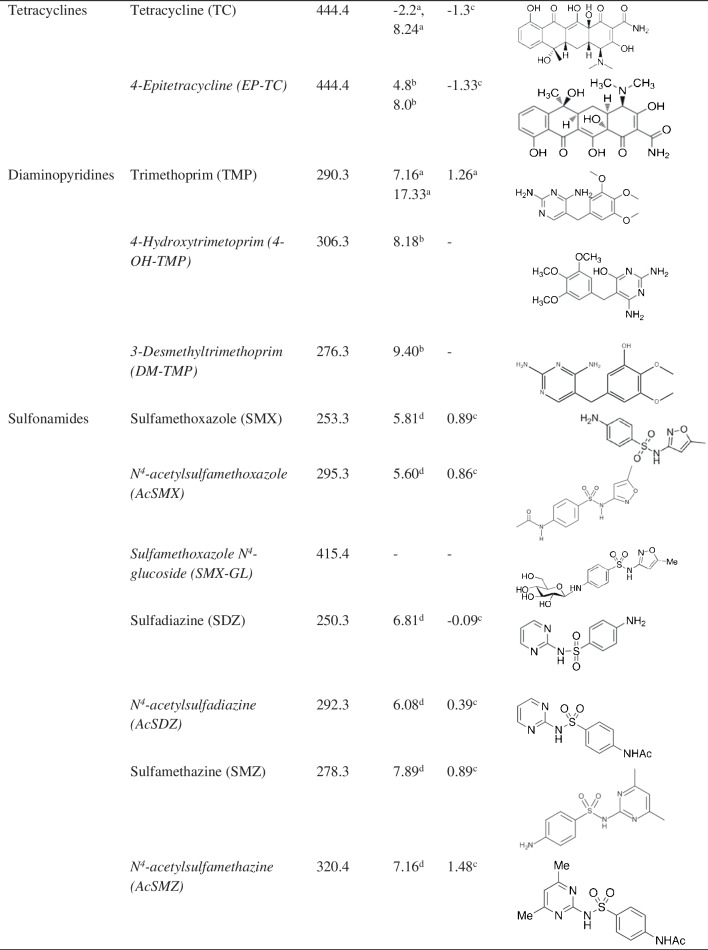
Classification and physical-chemical properties of the target compounds

Metabolites are indicated in italics; abbreviations are written in brackets. ^a^https://go.drugbank.com/; ^b^https://www.chemicalbook.com/; ^c^Li et al. [29]; ^d^Yuan et al. [30]

### Sample collection and pretreatment

Primary, secondary, and anaerobically digested and dehydrated sludge samples were collected from urban WWTPs. Compost samples were collected in a 1-year sampling period collected from a composting plant where anaerobically digested sludge from four urban WWTPs was treated in thermally controlled dynamic batteries with aeration provided by turning. Soil samples were Mediterranean type and were collected from three agricultural lands. Samples were freeze-dried in a Cryodos-50 lyophiliser (Telstar, Spain), homogenised in a mortar, and sieved (particle size < 100 mm).

### Sample treatment

Lyophilised samples [1.0 g dry weight (dw)] were transferred to glass centrifuge tubes and were spiked with the surrogate standards at 100 ng g^−1^ dw each. MeOH (3 mL) containing formic acid (0.5% v/v) was added to the tubes. Tubes were briefly vortex-mixed and sonicated in an ultrasonic bath for 15 min. After extraction, tubes were centrifuged for 10 min at 2900×*g*. The liquid phase was transferred to a clean tube whereas the solid phase was subjected to other two extraction cycles. Liquid phases from the three extraction cycles were combined in a glass centrifuge tube where 0.4 g of C18 were added for dispersive solid-phase extraction (d-SPE) clean-up. Tubes were vigorously shaken in a vortex-mixer for homogenization and centrifuged for 15 min at 2900×*g* for phase separation. The liquid phase was transferred to another tube and evaporated to dryness under a gentle nitrogen stream in a XcelVap^®^ automated evaporation system (NH, USA). The dried extract was dissolved in 0.3 mL of MeOH:water solution (1:1, v/v), filtered through a 0.22 µm cellulose syringe filter and collected in an automatic injector vial for LC–MS/MS determination.

### LC–MS/MS determination

Chromatographic determination was performed in an Agilent 1290 Infinity II chromatograph (Agilent, USA) equipped with a vacuum degasser, a binary pump, and an automatic injector. Chromatographic separation was carried out in a Zorbax RRHD Eclipse Plus C18 (150 mm × 3.0 mm i.d., 1.8 μm particle size) column (Agilent, USA), thermostated at 35 °C and protected with a Zorbax RRHD Eclipse Plus C18 (3.0 mm i.d., 1.8 µm particle size) guard column (Agilent, USA). Injection volume was 10 µL. Chromatographic conditions were those previously optimised and reported [31]. Briefly, MS/MS parameters were optimised in both positive and negative modes by direct infusion of 1 µg mL^−1^ individual and mixture solutions of the target compounds. Acetonitrile and MeOH were tested as organic solvents. Ammonium formate (with and without formic acid) and ammonium acetate (with and without acetic acid) aqueous solutions at different concentrations were tested as aqueous phase. The best results were obtained in positive mode. LC–MS/MS optimised conditions were: gradient elution with a flow rate of 0.4 mL min^−1^ using a mobile phase composed of 10 mM ammonium formate (0.05% v/v, formic acid) and MeOH. Elution started with 5% of MeOH, held for 1 min, increased to 30% in 3 min, then to 60% in 8 min, and finally to 100% in 2 min, and held for 2 min. Returning to initial conditions was performed in 2 min, held for 2 min for re-equilibration. The LC system was coupled to a 6495 triple quadrupole (QQQ) mass spectrometer (Agilent, USA) equipped with an electrospray ionisation source. MS parameters were as follows: capillary voltage, 4000 V; fragmentor, 166 V; nebuliser pressure, 40 psi; sheath gas temperature, 250 ºC; sheath gas flow rate, 12 L min^−1^; gas temperature, 350 °C; and drying gas flow rate, 11 L min^−1^. The analysis was carried out in dynamic multiple reaction monitoring mode (dMRM) operating in positive mode. The two most abundant transitions were monitored for each analyte. The most abundant transition was used for quantification and the other for confirmation. LC–MS/MS parameters for each compound are given in Table S1. Some authors have reported the formation of protomers of certain antibiotics, such as fluoroquinolones, during ionisation. This is due to the addition of protons in their multiple basic sites. The sample matrix can condition the protonation site preference and, therefore, affect the formation of different product ions [32]. To detect the formation of protomers, ion mobility separation coupled to high-resolution mass spectrometry (IMS-HRMS) has been reported to be pivotal [32]. As no IMS-HRMS instrument was available in our laboratory, we could not test the formation of protomers. Nevertheless, we checked that the ion ratio of product ions of each target compound was not affected by sample matrix. Instrument control and data acquisition were carried out with MassHunter software (Agilent, USA).

## Results and discussion

### Method optimisation

Method optimisation was carried out with compost sample aliquots (1.0 g dw) spiked with the target compounds at 100 ng g^−1^ dw. Experiments were performed in triplicate. Non-spiked compost matrix aliquots were also processed for blank correction. Compost samples were selected for method optimisation because their intermediate complexity with respect to the five types of solid matrices considered in this work. After method optimisation using compost samples, the method was validated for primary, secondary and digested sludge, compost and soil matrices. The type of extraction solvent, extraction solvent volume, extraction time, number of extraction cycles and the type and amount of disperser sorbent used for d-SPE extract clean-up were optimised. The initial conditions for method optimisation were fixed at 5 mL of extraction solvent, 10 min of extraction time, one extraction cycle and 0.8 g of C18 as d-SPE sorbent.

#### Extraction solvent optimisation

**Fig. 1:**
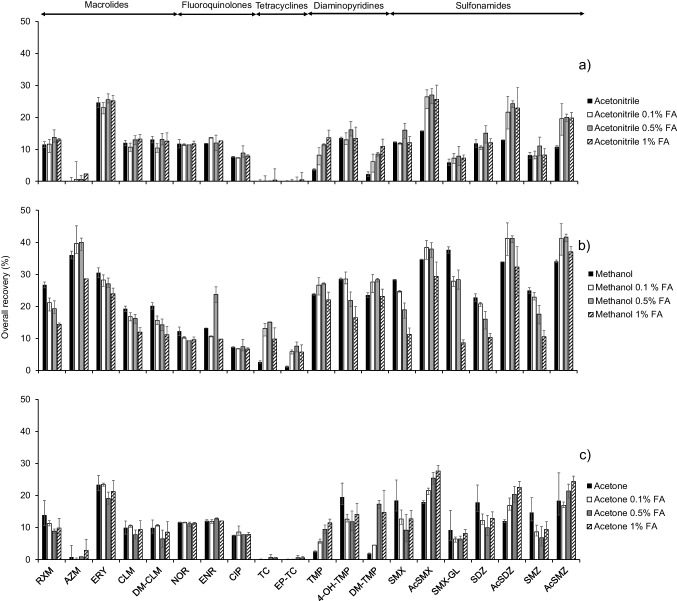
Overall recoveries obtained from (**a**) acetonitrile, (**b**) methanol and (**c**) acetone, pure and with different proportions of formic acid (FA)

Because of the significantly different physical–chemical properties of the target compounds (Table 1), two aprotic solvents (acetonitrile and acetone) and a protic solvent (MeOH) were evaluated as extraction solvents. They were tested as pure solvents and acidified with formic acid at 0.1% v/v, 0.5% v/v and 1% v/v. As can be seen in Fig. 1, the best extraction recoveries were obtained when MeOH was used as extraction solvent. The addition of 0.1% v/v or 0.5% v/v of formic acid to MeOH increased the extraction recoveries of most of the compounds whereas a higher formic acid content (1%, v/v) decreased the recoveries of all the antibiotics. Only fluoroquinolones were poorly affected by formic acid content. Tetracyclines (TC and EP-TC) were the antibiotics most influenced by the addition of formic acidic. This fact can be explained by their low p*K*_a_ values (-2.2 and 4.8, respectively). The addition of formic acid eases their presence in their unionised form and, therefore, their transference from the solid matrix to the extraction solvent increasing their recoveries. From these results, MeOH containing 0.5% v/v of formic acid was selected as extraction solvent for further experiments as this mixture provided the best average results.

#### Clean-up optimisation

**Fig. 2:**
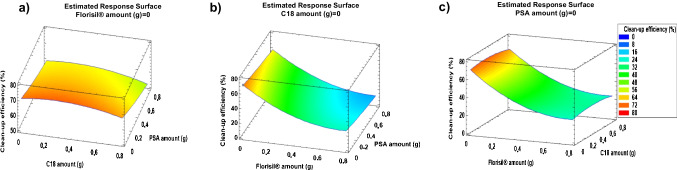
Response surface plots corresponding to clean-up efficiency versus (**a**) C18 and PSA amounts; (**b**) PSA and Florisil^®^ amounts; (**c**) C18 and Florisil^®^ amounts

Three sorbents (PSA, C18 and Florisil^®^) were tested for extract clean-up. A Box–Behnken design (BBD) was used for d-SPE optimisation for proper evaluation of the influence of each variable and their interactions. The number of experiments (*N*) required for BBD optimisation is given by the equation *N* = 2k(k − 1) + C_0_, where k is the number of variables and C_0_ is the number of central points [33]. A total of three variables were used, as they correspond to the types of dispersive sorbents evaluated, and the number of central points was set at 3. Therefore, the number of experiments for the simultaneous optimisation of the type and amount of disperser/s sorbent/s was 15. Each sorbent was tested at three levels (0, 0.4 and 0.8 g). Experiments were randomly performed. Table S2 displays the values set for each variable in each experiment. Statgraphics Centurion 18 software (StatPoint Technologies, Inc., Warrenton, VA, USA) was used for statistical data treatment. Experiments were carried out with sample extracts obtained from a compost sample. Compost aliquots (1.0 g dw) were extracted by sonication for 10 min with 5 mL of MeOH containing formic acid (0.5%, v/v). Extracts were spiked with 100 µL of a standard solution of the target compounds at a concentration of 1 µg mL^−1^ (each compound). The spiked extract concentration corresponds to a compost sample containing 100 ng g^−1^ dw of each compound. Clean-up efficiency was calculated as the ratio between the signals obtained from spiked extracts in comparison to the signals of the compounds in a MeOH:water (1:1, v/v) standard solution at the same concentration. Figure 2 displays the response surface plots corresponding to the geometric mean relative signals. The addition of Florisil^®^ caused a significant decrease on relative signals (Fig. 2b and c) which may be due to the removal of the target compounds by sorption onto Florisil^®^ sorbent. The addition of PSA also decreased relative signals (Fig. 2a) but not as significantly as when Florisil^®^ was used. For this reason, the use of Florisil^®^ and PSA sorbents was discarded, and extract clean-up by addition of C18 (0.4 g) (Fig. 2a and c) was selected for further studies.

#### UAE optimisation

**Fig. 3:**
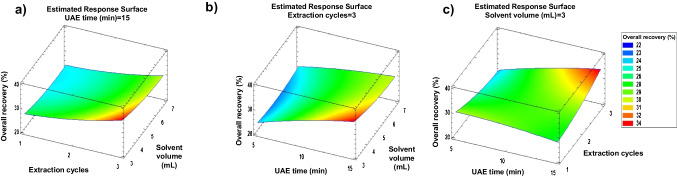
Response surface plots corresponding to matching to overall recovery versus (**a**) number of extraction cycles and solvent volume, (**b**) extraction time and solvent volume, (**c**) extraction time and number of extraction cycles

Once the extraction solvent was selected, the extraction solvent volume, extraction time and number of extraction cycles were optimised. These three variables (k = 3) were simultaneously optimised using a Box–Behnken design. The levels evaluated for each variable were as follows: extraction solvent volume: 3, 5 and 7 mL; extraction time: 5, 10 and 15 min; and number of extraction cycles: 1, 2 and 3. The number of central points was fixed at 3 (C_0_ = 3). Therefore, the number of experiments (*N* = 2k(k − 1) + C_0_) to carry out Box–Behnken design optimisation was 15. Values for each variable in each experiment can be seen in Table S3. Experiments were carried out with compost sample aliquots (1.0 g dw) spiked at 100 ng g^−1^ dw (each compound). After each experiment, extracts were subjected to clean-up by the addition of C18 (0.4 g). As can be seen in Fig. 3, the highest overall recoveries were obtained when three extraction cycles, 15 min of extraction time and 3 mL of extraction solvent were used. Therefore, these values were fixed at the optimum ones for further experiments. Figures S1 and S2 in Supplementary material display LC–MS/MS chromatograms of spiked and non-spiked sludge samples, respectively.

### Method validation

The method was validated for the determination of the target antibiotics and their metabolites in primary, secondary, and digested sludge, compost and soil. The validation was conducted in terms of linearity, method detection limits (MDLs), method quantification limits (MQLs), precision (expressed as relative standard deviation, RSD), absolute recovery (R) and accuracy (A), expressed as relative recovery.

The matrix effect (ME%) was evaluated at three concentration levels by comparing signals of the compounds in spiked sample extracts (A_spiked extract_), after blank correction (A_non-spiked extract_), and in standard solutions (A_standard_) at the same concentrations by applying the following Eq. (1):

1 $$\mathrm{ME }\;(\mathrm{\%}) = (({\mathrm{A}}_{\mathrm{spiked\; extract}}-{\mathrm{A}}_{\mathrm{non}\text{-}\mathrm{spiked \;extract}}-{\mathrm{A}}_{\mathrm{standard}})\times 100)/{\mathrm{A}}_{\mathrm{standard}}$$

A MeOH:water (1:1, v/v) solution was used as solvent for spiked extracts and standard solutions. Ion suppression was observed for all the compounds, mainly in primary and secondary sludge (Table 2). Macrolides and fluoroquinolones were the compounds with the highest signal suppression. For this reason, matrix-matched calibration standards were used for quantification. MDLs and MQLs were calculated from samples spiked at low concentration levels. They were set at concentrations providing signal-to-noise ratios of 3 and 10, respectively. MQLs for most of the compounds were lower than 7.5 ng g^−1^ dw in the four types of sludge and lower than 2.5 ng g^−1^ dw in soil with the exception, in all cases, of TC and EP-TC (MQLs between 12.5 and 25 ng g^−1^ dw) (Table 2). Eight-point matrix-matched calibration curves were prepared by spiking lyophilised samples (1.0 g dw) in triplicate with the target compounds at concentration levels from MQLs to 150 ng g^−1^ dw and with the surrogate standards at 100 ng g^−1^ dw each. Curves were obtained using analyte/surrogate peak area ratio versus analyte concentration. As the same concentration of surrogate standards was added to standard solutions and samples, analyte concentrations, instead of analyte/surrogate concentrations ratios, were used for curve construction. Curves were linear in the evaluated concentration range with correlation coefficients (R^2^) higher than or equal to 0.98 for all the compounds and matrices (Tables S4-S8 in Supplementary material).

**Table 2:** Average matrix effect (ME) and method detection (MDL) and quantification limits (MQL)

Compound	Primary sludge			Secondary sludge			Digested sludge			Compost			Soil		
	ME (%)	MDL (ng g^−1^)	MQL (ng g^−1^)	ME (%)	MDL (ng g^−1^)	MQL (ng g^−1^)	ME (%)	MDL (ng g^−1^)	MQL (ng g^−1^)	ME (%)	MDL (ng g^−1^)	MQL (ng g^−1^)	ME (%)	MDL (ng g^−1^)	MQL (ng g^−1^)
RXM	−94.6	1.50	5.00	−93.5	1.50	5.00	−94.9	1.50	5.00	−79.9	0.005	0.03	−3.45	0.002	0.05
AZM	−95.2	0.75	2.50	−93.1	0.75	2.50	−18.8	0.75	2.50	−24.3	0.05	0.50	−5.02	0.05	0.50
ERY	−97.3	5.00	7.50	−80.8	5.00	7.50	−95.8	5.00	7.50	−77.9	0.005	0.03	−49.2	0.005	0.03
CLM	−97.7	0.05	0.50	−92.3	0.05	0.50	−95.1	0.05	0.50	−78.6	0.005	0.03	−46.1	0.002	0.05
*DM-CLM*	−97.8	0.75	2.50	−86.9	0.75	2.50	−97.1	0.75	2.50	−84.8	0.005	0.03	−48.4	0.002	0.05
NOR	−97.6	0.05	0.50	−90.5	0.05	0.50	−96.5	0.05	0.50	−70.1	0.05	0.50	−41.5	0.05	0.50
ENR	−90.9	0.05	0.50	−99.5	0.05	0.50	−82.1	0.05	0.50	−65.2	0.05	0.50	−50.4	0.05	0.50
CIP	−92.2	0.05	0.50	−90.5	0.05	0.50	−92.7	0.05	0.50	−63.9	0.05	0.50	−35.3	0.05	0.50
TC	−84.0	12.5	25.0	−77.4	12.5	25.0	−36.4	12.5	25.0	−4.33	12.5	25.0	−5.08	5.00	12.5
*EP-TC*	−48.7	12.5	25.0	−43.3	12.5	25.0	−13.0	12.5	25.0	−3.41	12.5	25.0	−4.03	12.5	25.0
TMP	−93.8	0.05	0.50	−97.8	0.05	0.50	−80.1	0.05	0.50	−37.2	0.05	0.50	−2.24	0.05	0.50
*4-OH-TMP*	−72.5	0.75	2.50	−67.8	0.75	2.50	−38.1	0.75	2.50	−7.62	5.00	7.50	−2.80	1.50	5.00
*DM-TMP*	−63.5	0.002	0.05	−42.2	0.002	0.05	−54.9	0.002	0.05	−52.0	0.005	0.03	−3.15	0.002	0.05
SMX	−62.1	0.005	0.03	−54.1	0.005	0.03	−51.8	0.005	0.03	−15.1	0.005	0.03	−1.42	0.001	0.03
*AcSMX*	−78.4	0.005	0.03	−70.8	0.005	0.03	−73.4	0.005	0.03	−15.1	0.005	0.03	−1.21	0.001	0.03
*SMX-GL*	−48.6	5.00	7.50	−16.2	5.00	7.50	−13.4	5.00	7.50	−15.4	5.00	7.50	−8.90	0.75	2.50
SDZ	−75.3	0.75	2.50	−69.2	0.75	2.50	−75.3	0.75	2.50	−24.2	0.002	0.05	−1.17	0.05	0.50
*AcSDZ*	−48.6	0.005	0.03	−49.4	0.005	0.03	−42.2	0.005	0.03	−42.2	0.05	0.50	−3.39	0.05	0.50
SMZ	−57.0	0.005	0.03	−31.4	0.005	0.03	−43.5	0.005	0.03	−5.08	0.005	0.03	−6.19	0.002	0.05
*AcSMZ*	−16.8	0.005	0.03	−13.4	0.005	0.03	−8.04	0.005	0.03	−5.74	0.005	0.03	−2.16	0.001	0.03

Metabolites are indicated in italics. MDL and MQL values refer to dry weight

Extraction recovery, accuracy and precision of the method were evaluated by spiking lyophilised samples with the target compounds at three concentration levels and with the surrogate standards at 100 ng g^−1^ dw each. Experiments were carried out in triplicate Spike concentrations were 1.5 ng g^−1^ dw, 22.5 ng g^−1^ dw and 45 ng g^−1^ dw, except for 4-OH-TMP, SMX-GL and EP-TC that were spiked at higher concentration levels (see detailed information in Tables S4–S8) due to their higher MQL values. Extraction recoveries (R) were calculated by comparison of the peak areas obtained from spiked samples (A_spiked sample_) with those from spiked extracts (A_spiked extract_), after blank correction (A_non-spiked sample_), applying Eq. (2):

2 $$\mathrm{R }\;(\mathrm{\%}) = \left({\mathrm{A}}_{\mathrm{spiked\; sample}}-{\mathrm{A}}_{\mathrm{non}\text{-}\mathrm{spiked\; sample}}\right)\times 100/\left({\mathrm{A}}_{\mathrm{spiked\; extract}}-{\mathrm{A}}_{\mathrm{non}\text{-}\mathrm{spiked\; sample}}\right)$$

Accuracy (A), expressed as relative recovery, was determined by comparison of the concentrations obtained from spiked samples using matrix-matched calibration curves (C_spiked sample_), after blank correction (C_non-spiked sample_), with the spike concentration (C_spike concentration_) applying Eq. (3):

3 $$\mathrm{A }\;(\mathrm{\%}) =\left({\mathrm{C}}_{\mathrm{spiked\; sample}}-{\mathrm{C}}_{\mathrm{non}\text{-}\mathrm{spiked\; sample}}\right)\times 100/{\mathrm{C}}_{\mathrm{spike\; concentration}}$$

Precision was calculated as inter-day repeatability and expressed as relative standard deviation (RSD). Average extraction recovery, accuracy, and precision values for each type of sample can be seen in Table 3. Mean accuracy values were in the range of 69.7–106% for macrolides, 88.7–101% for fluoroquinolones, 78.3–97.7% for tetracyclines, 80.4–105% for diaminopyridines and 82.1–103% for sulfonamides. RSD values were below 17% for all compounds in the five environmental solid matrices. Detailed results obtained for each concentration level and sample matrix can be seen in Tables S4–S8. Despite the high matrix effect values obtained for macrolides and fluoroquinolones in primary, secondary and digested sludge, good accuracy and precision values were obtained for such compounds and matrices [accuracy (%): 69.7–101, with RSD values from 3.1 to 15% (Table 3)], using matrix-matched calibration curves and surrogate standards. Method robustness was evaluated to ensure that the matrix nature did not affect the appropriate performance of the method. Five different matrices of each type of sample, i.e. five different primary sludges, five different secondary sludges, etc., were spiked at three concentration levels. Non-spiked samples were also processed for blank correction. Experiments were carried out in triplicate. Concentrations found at each spike level and type of sample were statistically compared with the spike concentration using Student’s *t*-test at a 95% confidence level. The robustness of the method was also assessed using the RSD values for each type of matrix and spike concentration. Robustness assessment results for each type of matrix can be seen in Tables S9–S13 in Supplementary material. The absolute values of the calculated *t* were lower than the critical value [|*t*_4_|: 2.78 (*P* = 0.05)] for all the compounds, samples, and spike levels except for some compounds and spike levels in primary sludge (RXM and DM-CLM at 10 ng g^−1^ dw and ERY at 22.5 and 45 ng g^−1^ dw) and secondary sludge (AZM at 22.5 ng g^−1^ dw). Therefore, the null hypothesis of not significant difference between the obtained concentrations and the spike concentrations cannot be rejected with a 95% level of confidence. For most of the compounds, samples and spike levels, RSD values were lower than 15%, except for primary sludge where most RSD values were up to 20% (Tables S9-S13 in Supplementary material). The highest relative standard deviation was 28% and corresponded to CLM and DM-CLM in primary sludge, which is the most complex matrix, at the low spike level. These values are comparable to or lower than those reported by other authors for the determination of antibiotics in soil [16] and sludge [22]. For instance, Ajibola et al. [22] reported average RSD values lower than 20% but up to 24%, 29% and 32% for ERY, SMX and CLM, respectively. The method has been proven to be robust enough through different samples.

**Table 3:** Recovery (R), accuracy (A) and precision of the method, expressed as relative standard deviation (RSD)

Compound	Primary sludge			Secondary sludge			Digested sludge			Compost			Soil		
	R (%)	A (%)	RSD (%)	R (%)	A (%)	RSD (%)	R (%)	A (%)	RSD (%)	R (%)	A (%)	RSD (%)	R (%)	A (%)	RSD (%)
RXM	35.8	70.5	14	46.9	93.7	7.9	79.8	95.0	6.0	83.4	94.2	9.4	88.7	97.7	2.6
AZM	65.0	69.7	14	84.3	86.6	7.5	93.5	91.8	10	89.4	99.1	13	98.3	99.9	3.1
ERY	42.9	76.0	12	71.9	80.6	6.2	77.9	86.2	9.6	81.8	97.6	14	93.1	99.8	1.7
CLM	14.4	79.2	10	47.9	84.4	15	65.7	101	5.9	67.4	106	12	80.3	100	2.7
*DM-CLM*	44.0	76.8	13	59.4	83.6	8.5	54.4	90.3	6.9	81.4	101	13	92.6	101	2.9
NOR	14.0	86.1	11	32.1	80.7	9.5	42.3	90.5	13	43.8	90.2	5.1	95.5	101	3.4
ENR	22.2	87.2	14	36.8	87.6	13	37.1	91.1	2.7	38.3	88.8	4.8	76.3	100	10
CIP	16.7	80.1	14	17.3	89.2	9.0	25.3	86.2	3.1	41.4	92.0	4.6	48.3	97.3	1.6
TC	12.2	80.6	14	12.9	79.7	9.5	16.2	91.2	10	16.6	95.9	17	17.0	87.8	12
*EP-TC*	9.38	80.4	15	9.39	78.3	5.3	11.0	85.9	11	11.2	87.8	11	55.4	97.7	9.8
TMP	30.0	91.2	12	30.6	105	7.6	43.9	101	3.0	58.6	99.1	2.4	98.3	99.1	2.6
*4-OH-TMP*	32.1	88.7	12	40.6	88.9	4.6	43.4	96.5	3.2	66.4	97.4	5.5	76.9	96.8	11
*DM-TMP*	22.2	97.4	14	30.3	93.4	8.0	44.0	97.9	3.3	47.6	99.2	3.6	59.3	98.0	2.0
SMX	38.1	88.4	15	38.5	97.8	4.3	41.7	96.8	3.8	46.2	97.6	6.7	76.7	101	10
*AcSMX*	43.0	87.4	14	86.3	95.1	4.6	86.3	102	3.6	89.1	99.6	8.0	91.9	99.4	2.1
*SMX-GL*	29.1	90.6	16	37.1	90.8	5.1	51.3	90.7	13	56.1	97.6	17	86.0	100	16
SDZ	31.5	94.6	12	34.5	101	5.1	36.4	102	3.3	42.8	103	14	83.0	97.3	9.8
*AcSDZ*	51.1	83.4	13	42.8	82.1	4.2	76.5	90.7	4.5	82.0	98.1	5.4	92.0	98.3	5.9
SMZ	32.7	95.5	13	38.6	99.5	4.4	41.7	101	2.2	44.5	95.6	6.1	64.2	98.3	8.1
*AcSMZ*	62.4	86.2	15	71.0	86.5	1.9	85.7	92.9	3.3	85.7	102	6.8	97.6	98.8	1.5

Metabolites are indicated in italics

## Method application

The applicability of the proposed method was evaluated by the determination of the target compounds in primary sludge (*n* = 3), secondary sludge (*n* = 3) and anaerobically digested and dehydrated sludge (*n* = 3) samples collected from three anaerobic urban WWTPs, a compost sample and soil samples (*n* = 3) from three agricultural lands. Obtained results can be found in Table S14. Macrolides and fluoroquinolones were the antibiotics most frequently detected (100% of analysed samples) and those at the highest concentration levels. The highest concentrations were measured in primary sludge [up to 193 ng g^−1^ dw for macrolides (RXM) and up to 199 ng g^−1^ dw for fluoroquinolones (CIP)] and in secondary sludge [up to 185 ng g^−1^ dw for macrolides (AZM) and up to 291 ng g^−1^ dw for fluoroquinolones (CIP)]. Their concentrations decreased after digestion [up to 96.9 ng g^−1^ dw (RXM)] and in compost (up to 27.0 ng g^−1^ dw (NOR) and soil samples [up to 92.9 ng g^−1^ dw (DM-CLM)]. The same effect was observed for diaminopyridines and sulfonamides. They were detected in primary and secondary sludge (up to 78.1 ng g^−1^ dw corresponding to SMZ and up to 91.0 ng g^−1^ dw corresponding to SMX) but they were rarely detected in compost and soil samples, and when detected, their concentrations were lower than 0.6 ng g^−1^ dw. In six of the analysed samples the concentrations of some metabolites of CLM (DM-CLM), TMP (4-OH-TMP and DM-TMP) and SMZ (AcSMZ) were higher than those of their parent compounds. The same effect was reported by García-Galán [15] for AcSMZ (*n* = 17; frequency of detection: 24%; mean value: 9.81 ng g^−1^) in comparison to its parent compound SMZ in sewage sludge (*n* = 17; frequency of detection: 30%; mean value: 1.7 ng g^−1^). The other results cannot be compared with data from literature because, to our knowledge, this is the first study reporting concentrations of antibiotics and their metabolites in primary and secondary sludge and the first one for the determination of metabolites of CLM, TC and TMP in both non-treated and treated sludge and soil.

## Conclusions

A method has been optimised and validated for the first time for determining the presence of five critically and highly important classes of antibiotics and eight of their metabolites in non-treated sewage sludge (primary and secondary sludge), treated sludge (digested and composted sludge) and agricultural soil. Sample extraction and extract clean-up are based on easy-to-perform and low-cost techniques (UAE and d-SPE), making the method suitable for routine control of the presence of target antibiotics in agricultural soils and sludge. MQL values were in the range of 0.03–7.5 ng g^−1^ dw, except for TC and EP-TC (up to 25 ng g^−1^ dw). Precision, expressed as relative standard deviation, was lower than 17% for all the compounds (mean value: 8.3%). Average accuracy, expressed as relative recovery, was in the range of 69.7–106% (mean value: 92.7%). The method was proven to be sufficiently robust through different samples. The application of the method revealed that macrolides and fluoroquinolones were the antibiotics at the highest concentrations and that some of the metabolites were at similar or higher concentrations than their parent compounds in some of the analysed samples. The proposed method can provide a useful tool to (i) obtain information about the occurrence and fate of high concern classes of antibiotics and their metabolites in sludge treatment process, (ii) evaluate their presence in treated sludge prior to its application on agricultural soils as fertiliser, and (iii) evaluate their occurrence and fate in agricultural soils. Further studies should be carried out to evaluate the presence of selected classes of antibiotics in reclaimed wastewater and/or surface water, as they could constitute other means for the release of antibiotics into agricultural soils.

### Supplementary Information

Below is the link to the electronic supplementary material.

Supplementary file1 (DOCX 455 KB)
