# Supplementary material for: Multiresidue method for the determination of critically and highly important classes of antibiotics and their metabolites in agricultural soils and sewage sludge
Source: Anal Bioanal Chem. 2023 Oct 17;415(29-30):7161–73. doi: 10.1007/s00216-023-04982-3 (PMC10684426; doi:10.1007/s00216-023-04982-3)
Supplement: Supplementary file 1 — Supplementary file1 (DOCX 455 KB) [file 216_2023_4982_MOESM1_ESM.docx]

Supplementary material

**Multiresidue method for the determination of critically and highly important classes of antibiotics and their metabolites in agricultural soils and sewage sludge**

Carmen Mejías, Juan Luis Santos, Julia Martín, Irene Aparicio, Esteban Alonso*

Departamento de Química Analítica, Escuela Politécnica Superior, Universidad de Sevilla. 41011 Seville, Spain.

*Corresponding autor

*Address*:

Departamento de Química Analítica

Escuela Politécnica Superior

Universidad de Sevilla

C/ Virgen de África, 7

41011 Seville (Spain)

*E-mail address*: jlsantos@us.es

**Table S1.** LC-MS/MS parameters for the determination of the target compounds.

| Antibiotic/  metabolite | Surrogate standard | Precursor ion *(m/z)* | Product ions (quantifier/  qualifier) *(m/z)* | CE  (eV) | Ion ratio | Retention time (min) |
| --- | --- | --- | --- | --- | --- | --- |
| Macrolides |  |  |  |  |  |  |
| RXM | ERY-^13^C | 838.1 | 158.1/679.4 | 32/20 | 72.9 | 15.76 |
| AZM | ERY-^13^C | 750.0 | 591.5/116.1 | 28/44 | 56.1 | 12.35 |
| ERY | ERY-^13^C | 734.5 | 83.0/576.4 | 68/20 | 81.4 | 15.71 |
| CLM | ERY-^13^C | 749.0 | 158.1/590.4 | 28/16 | 49.5 | 15.72 |
| *DM-CLM* | ERY-^13^C | 734.9 | 144.1/576.4 | 24/16 | 23.5 | 15.73 |
| Fluoroquinolones |  |  |  |  |  |  |
| NOR | OFL-d_3_ | 320.3 | 302.1/231.0 | 24/44 | 26.0 | 8.56 |
| ENR | OFL-d_3_ | 360.4 | 286.1/342.1 | 40/40 | 61.7 | 9.08 |
| CIP | OFL-d_3_ | 332.1 | 314.1/231.0 | 16/40 | 98.5 | 8.79 |
| Tetracyclines |  |  |  |  |  |  |
| TC | OFL-d_3_ | 445.4 | 410.2/154.1 | 20/28 | 57.9 | 9.05 |
| *EP-TC* | OFL-d_3_ | 445.4 | 410.2/98.1 | 20/48 | 27.0 | 8.13 |
| Diaminopyridines |  |  |  |  |  |  |
| TMP | SMX-^13^C | 291.2 | 261.1/229.8 | 28/24 | 98.2 | 7.79 |
| *4-OH-TMP* | SMX-^13^C | 279.2 | 93.0/121.1 | 40/40 | 1.10 | 8.27 |
| *DM-TMP* | SMX-^13^C | 277.3 | 261.4/123.0 | 28/44 | 63.1 | 6.80 |
| Sulfonamides |  |  |  |  |  |  |
| SMX | SMX-^13^C | 254.3 | 92.1/108.0 | 28/28 | 76.1 | 8.96 |
| *AcSMX* | SMX-^13^C | 296.3 | 134.0/108.1 | 24/28 | 49.8 | 10.74 |
| *SMX-GL* | SMX-^13^C | 416.4 | 254.0/108.0 | 8/44 | 9.50 | 7.59 |
| SDZ | SMX-^13^C | 251.3 | 92.1/156.0 | 28/12 | 98.0 | 6.45 |
| *AcSDZ* | SMX-^13^C | 293.3 | 134.1/198.0 | 24/16 | 74.9 | 7.71 |
| SMZ | SMX-^13^C | 279.3 | 186.0/92.0 | 16/36 | 76.4 | 8.28 |
| *AcSMZ* | SMX-^13^C | 321.4 | 186.0/134.0 | 20/28 | 81.3 | 9.09 |
| Surrogate standards | | | | | | |
| ERY-^13^C | - | 736.9 | 160.1/578.4 | 32/16 | 56.2 | 15.31 |
| OFL-d_3_ | - | 365.4 | 321.2/261.1 | 20/28 | 90.7 | 8.25 |
| SMX-^13^C | - | 260.2 | 98.1/162.0 | 32/16 | 94.5 | 8.95 |

CE: collision energy; Metabolites are written in cursive font.

**Table S2.** Box–Behnken design matrix for the optimisation of clean-up sorbent type and amount.

| **Experiment** | **C18**  **amount (g)** | **PSA**  **amount (g)** | **Florisil® amount (g)** |
| --- | --- | --- | --- |
| 1 | - | 0.4 | - |
| 2 | 0.4 | - | 0.8 |
| 3 | 0.8 | 0.8 | 0.4 |
| 4 | - | 0.8 | 0.4 |
| 5 | 0.4 | 0.4 | 0.4 |
| 6 | - | - | 0.4 |
| 7 | 0.4 | 0.8 | 0.8 |
| 8 | - | 0.4 | 0.8 |
| 9 | 0.4 | - | - |
| 10 | 0.8 | - | 0.4 |
| 11 | 0.4 | 0.4 | 0.4 |
| 12 | 0.4 | 0.4 | 0.4 |
| 13 | 0.8 | 0.4 | - |
| 14 | 0.8 | 0.4 | 0.8 |
| 15 | 0.4 | 0.8 | - |

**Table S3.** Box–Behnken design matrix for the optimisation of extraction solvent volume, time of extraction and number of extraction cycles.

| **Experiment** | **Solvent volume (mL)** | **Extraction time (min)** | **Number of extractions cycles** |
| --- | --- | --- | --- |
| 1 | 3 | 15 | 2 |
| 2 | 5 | 10 | 2 |
| 3 | 3 | 10 | 1 |
| 4 | 5 | 5 | 3 |
| 5 | 5 | 15 | 1 |
| 6 | 3 | 10 | 3 |
| 7 | 7 | 10 | 3 |
| 8 | 5 | 10 | 2 |
| 9 | 5 | 5 | 1 |
| 10 | 5 | 15 | 3 |
| 11 | 5 | 10 | 2 |
| 12 | 7 | 15 | 2 |
| 13 | 7 | 5 | 2 |
| 14 | 3 | 5 | 2 |
| 15 | 7 | 10 | 1 |

**Table S4.** Recovery (R), accuracy (A), matrix effect (ME), precision of the method expressed as relative standard deviation (RSD) and matrix-matched calibration curves correlation coefficients (R^2^) for primary sludge.

| Compound | Low level (1.5 ng g^-1^ dw) | | | | Medium level (22.5 ng g^-1^ dw) | | | | High level (45 ng g^-1^ dw) | | | | R^2^ |
| --- | --- | --- | --- | --- | --- | --- | --- | --- | --- | --- | --- | --- | --- |
|  | R (%) | A (%) | ME (%) | RSD (%) | R (%) | A (%) | ME (%) | RSD  (%) | R (%) | A  (%) | ME (%) | RSD (%) |  |
| RXM^1^ | 37.2 | 77.8 | -95.3 | 14 | 36.7 | 63.6 | -99.2 | 17 | 33.6 | 70.1 | -89.2 | 11 | 0.989 |
| AZM^1^ | 69.1 | 68.9 | -91.1 | 10 | 60.1 | 78.1 | -95.7 | 18 | 65.7 | 62.1 | -98.8 | 14 | 0.964 |
| ERY^1^ | 44.1 | 74.9 | -97.0 | 16 | 44.4 | 79.5 | -99.5 | 15 | 40.3 | 73.6 | -95.3 | 3.6 | 0.987 |
| CLM | 15.0 | 75.4 | -95.1 | 14 | 15.0 | 92.3 | -98.8 | 13 | 13.1 | 70.0 | -99.2 | 3.7 | 0.992 |
| DM-CLM^1^ | 41.2 | 73.2 | -98.4 | 18 | 45.9 | 81.5 | -99.6 | 14 | 44.8 | 75.8 | -95.4 | 8.5 | 0.990 |
| NOR | 14.7 | 99.9 | -97.5 | 15 | 15.3 | 81.1 | -97.2 | 2.6 | 12.1 | 77.3 | -98.1 | 16 | 0.981 |
| ENR | 21.9 | 104 | -94.8 | 14 | 23.2 | 87.8 | -93.0 | 11 | 21.6 | 69.7 | -84.9 | 16 | 0.983 |
| CIP | 18.0 | 99.1 | -89.4 | 12 | 12.8 | 71.3 | -92.5 | 19 | 19.4 | 69.9 | -94.7 | 10 | 0.987 |
| TC^2^ | 13.8 | 95.8 | -81.9 | 13 | 11.7 | 70.2 | -86.3 | 14 | 11.0 | 75.9 | -83.9 | 15 | 0.967 |
| EP-TC^2^ | 9.32 | 99.1 | -58.4 | 14 | 8.87 | 69.9 | -48.9 | 14 | 9.96 | 72.1 | -38.9 | 17 | 0.989 |
| TMP | 31.1 | 100 | -95.6 | 17 | 29.5 | 96.2 | -94.2 | 6.2 | 29.3 | 77.5 | -91.6 | 13 | 0.993 |
| 4-OH-TMP^1^ | 30.9 | 98.9 | -72.1 | 17 | 32.1 | 94.1 | -72.9 | 11 | 33.3 | 73.1 | -72.5 | 10 | 0.989 |
| DM-TMP | 21.0 | 99.5 | -67.9 | 19 | 23.9 | 93.8 | -60.9 | 7.2 | 21.8 | 99.0 | -61.8 | 16 | 0.997 |
| SMX | 41.3 | 101 | -62.7 | 15 | 37.6 | 75.5 | -61.6 | 7.6 | 35.3 | 88.8 | -62.0 | 22 | 0.994 |
| AcSMX | 37.8 | 96.7 | -82.4 | 12 | 40.1 | 79.8 | -78.5 | 16 | 51.0 | 85.8 | -74.3 | 12 | 0.994 |
| SMX-GL^1^ | 30.3 | 98.6 | -44.9 | 14 | 30.1 | 92.5 | -56.0 | 16 | 26.9 | 80.8 | -44.8 | 19 | 0.993 |
| SDZ^1^ | 31.8 | 99.7 | -77.0 | 15 | 28.7 | 90.3 | -76.0 | 12 | 33.9 | 93.7 | -73.0 | 8.3 | 0.998 |
| AcSDZ | 48.8 | 96.9 | -48.3 | 17 | 49.7 | 74.5 | -47.5 | 12 | 54.8 | 78.9 | -50.0 | 11 | 0.993 |
| SMZ | 32.0 | 100 | -52.5 | 15 | 32.0 | 92.9 | -59.8 | 12 | 34.2 | 93.5 | -58.7 | 11 | 0.988 |
| AcSMZ | 62.7 | 104 | -15.0 | 19 | 57.5 | 80.0 | -18.4 | 13 | 67.0 | 74.6 | -17.1 | 12 | 0.991 |

^1^Spike levels: 10, 22.5 and 45 ng g^-1^ dw; ^2^Spike levels: 25, 35 and 45 ng g^-1^ dw; Parent compounds are marked in bold.

**Table S5.** Recovery (R), accuracy (A), matrix effect (ME), precision of the method expressed as relative standard deviation (RSD) and matrix-matched calibration curves correlation coefficients (R^2^) for secondary sludge.

| Compound | Low level (1.5 ng g^-1^ dw) | | | | Medium level (22.5 ng g^-1^ dw) | | | | High level (45 ng g^-1^ dw) | | | | R^2^ |
| --- | --- | --- | --- | --- | --- | --- | --- | --- | --- | --- | --- | --- | --- |
|  | R (%) | A (%) | ME (%) | RSD (%) | R (%) | A (%) | ME (%) | RSD  (%) | R (%) | A  (%) | ME (%) | RSD (%) |  |
| RXM^1^ | 44.9 | 97.8 | -92.0 | 5.8 | 47.5 | 78.2 | -90.0 | 7.1 | 48.3 | 105 | -98.5 | 11 | 0.989 |
| AZM^1^ | 95.0 | 100 | -90.9 | 9.7 | 87.7 | 69.5 | -91.2 | 4.5 | 70.3 | 90.4 | -97.2 | 8.5 | 0.964 |
| ERY^1^ | 73.5 | 88.6 | -85.6 | 2.9 | 72.9 | 80.2 | -82.9 | 14 | 69.4 | 73.1 | -73.8 | 2.0 | 0.987 |
| CLM | 47.4 | 83.9 | -96.4 | 16 | 49.8 | 74.8 | -90.0 | 10 | 46.5 | 94.6 | -90.5 | 19 | 0.992 |
| DM-CLM^1^ | 59.7 | 102 | -79.6 | 9.1 | 58.7 | 74.2 | -93.1 | 3.3 | 59.9 | 74.5 | -88.1 | 13 | 0.990 |
| NOR | 37.6 | 84.5 | -90.9 | 15 | 37.2 | 83.9 | -88.9 | 5.3 | 21.5 | 73.8 | -91.7 | 8.0 | 0.981 |
| ENR | 37.3 | 98.3 | -98.7 | 17 | 34.2 | 73.5 | -99.9 | 13 | 38.9 | 91.0 | -99.9 | 8.5 | 0.983 |
| CIP | 17.4 | 95.8 | -91.2 | 12 | 20.2 | 83.7 | -89.8 | 11 | 14.4 | 88.1 | -90.5 | 4.1 | 0.987 |
| TC^2^ | 10.2 | 76.3 | -74.6 | 6.4 | 12.6 | 75.7 | -83.2 | 16 | 15.9 | 87.2 | -74.3 | 6.2 | 0.967 |
| EP-TC^2^ | 9.76 | 87.3 | -42.2 | 9.0 | 8.99 | 73.7 | -41.7 | 0.2 | 9.43 | 74.0 | -46.0 | 6.6 | 0.989 |
| TMP | 27.3 | 111 | -96.7 | 8.4 | 29.8 | 107 | -98.8 | 7.0 | 34.8 | 96.1 | -98.0 | 7.6 | 0.993 |
| 4-OH-TMP^1^ | 40.9 | 87.1 | -67.8 | 7.3 | 44.5 | 95.5 | -74.2 | 4.0 | 36.5 | 84.2 | -61.6 | 2.3 | 0.989 |
| DM-TMP | 31.0 | 101 | -36.0 | 8.6 | 26.1 | 83.7 | -49.3 | 6.8 | 33.7 | 95.6 | -41.2 | 8.6 | 0.997 |
| SMX | 34.3 | 104 | -55.2 | 4.7 | 41.2 | 96.7 | -57.8 | 3.7 | 40.1 | 92.8 | -49.4 | 4.7 | 0.994 |
| AcSMX | 97.9 | 133 | -78.1 | 7.2 | 82.8 | 76.5 | -72.1 | 3.5 | 78.1 | 75.9 | -62.2 | 3.1 | 0.994 |
| SMX-GL^1^ | 32.5 | 92.7 | -10.1 | 5.6 | 41.1 | 90.2 | -28.3 | 8.1 | 37.7 | 89.6 | -10.1 | 1.6 | 0.993 |
| SDZ^1^ | 39.7 | 97.1 | -66.7 | 9.7 | 31.1 | 102 | -72.3 | 0.3 | 32.6 | 105 | -68.7 | 5.3 | 0.998 |
| AcSDZ | 44.6 | 98.9 | -40.9 | 8.3 | 43.1 | 75.7 | -45.9 | 1.3 | 40.6 | 71.7 | -61.4 | 2.9 | 0.993 |
| SMZ | 43.6 | 98.2 | -29.1 | 7.5 | 36.1 | 99.4 | -36.3 | 2.1 | 36.1 | 101 | -28.8 | 3.5 | 0.988 |
| AcSMZ | 69.3 | 97.3 | -15.5 | 4.4 | 81.2 | 78.3 | -18.1 | 0.2 | 62.5 | 84.0 | -6.70 | 1.2 | 0.991 |

^1^Spike levels: 10, 22.5 and 45 ng g^-1^ dw; ^2^Spike levels: 25, 35 and 45 ng g^-1^ dw; Parent compounds are marked in bold.

**Table S6.** Recovery (R), accuracy (A), matrix effect (ME), precision of the method expressed as relative standard deviation (RSD) and matrix-matched calibration curves correlation coefficients (R^2^) for digested sludge.

| Compound | Low level (1.5 ng g^-1^ dw) | | | | Medium level (22.5 ng g^-1^ dw) | | | | High level (45 ng g^-1^ dw) | | | | R^2^ |
| --- | --- | --- | --- | --- | --- | --- | --- | --- | --- | --- | --- | --- | --- |
|  | R (%) | A (%) | ME (%) | RSD (%) | R (%) | A (%) | ME (%) | RSD  (%) | R (%) | A  (%) | ME (%) | RSD (%) |  |
| RXM^1^ | 88.8 | 97.7 | -98.3 | 5.0 | 76.5 | 93.7 | -92.6 | 3.8 | 74.1 | 93.6 | -93.9 | 9.3 | 0.989 |
| AZM^1^ | 90.4 | 99.3 | -17.9 | 3.8 | 99.8 | 92.4 | -23.0 | 20 | 90.2 | 83.7 | -15.6 | 7.3 | 0.964 |
| ERY^1^ | 72.1 | 99.6 | -95.4 | 6.8 | 85.1 | 78.3 | -95.5 | 5.6 | 76.6 | 80.6 | -96.6 | 17 | 0.987 |
| CLM | 82.7 | 101 | -96.1 | 8.4 | 63.8 | 101 | -93.8 | 6.4 | 50.6 | 99.5 | -95.5 | 3.0 | 0.992 |
| DM-CLM^1^ | 62.8 | 93.0 | -97.9 | 9.2 | 50.7 | 94.3 | -96.3 | 6.7 | 49.7 | 83.6 | -97.2 | 4.9 | 0.990 |
| NOR | 59.1 | 82.9 | -97.0 | 3.0 | 42.6 | 97.5 | -95.9 | 28 | 25.2 | 91.1 | -96.7 | 8.7 | 0.981 |
| ENR | 39.5 | 102 | -77.1 | 1.9 | 40.6 | 83.1 | -83.0 | 1.7 | 31.2 | 88.2 | -86.2 | 4.4 | 0.983 |
| CIP | 20.9 | 94.8 | -95.1 | 1.3 | 29.7 | 83.5 | -93.1 | 5.9 | 25.4 | 80.3 | -90.0 | 1.9 | 0.987 |
| TC^2^ | 11.6 | 84.7 | -36.8 | 11 | 12.0 | 93.6 | -40.7 | 9.9 | 25.0 | 95.4 | -31.7 | 9.0 | 0.967 |
| EP-TC^2^ | 9.89 | 101 | -22.9 | 10 | 9.08 | 81.0 | -10.3 | 11 | 13.9 | 75.6 | -5.65 | 11 | 0.989 |
| TMP | 47.5 | 109 | -78.5 | 1.1 | 53.7 | 101 | -80.5 | 3.4 | 30.6 | 93.5 | -81.4 | 4.6 | 0.993 |
| 4-OH-TMP^1^ | 47.4 | 107 | -40.1 | 5.7 | 41.7 | 83.9 | -41.8 | 1.7 | 41.2 | 98.5 | -32.5 | 2.2 | 0.989 |
| DM-TMP | 42.1 | 93.7 | -54.6 | 3.3 | 43.1 | 92.1 | -54.4 | 3.4 | 46.8 | 108 | -55.7 | 3.1 | 0.997 |
| SMX | 42.0 | 95.8 | -55.1 | 9.1 | 36.8 | 93.5 | -46.9 | 0.7 | 46.2 | 101 | -53.5 | 1.5 | 0.994 |
| AcSMX | 97.9 | 98.3 | -67.0 | 3.2 | 82.8 | 101 | -74.1 | 3.7 | 78.1 | 107 | -79.2 | 3.8 | 0.994 |
| SMX-GL^1^ | 53.1 | 97.1 | -10.0 | 8.6 | 50.1 | 77.6 | -18.4 | 5.0 | 50.8 | 101 | -11.8 | 25 | 0.993 |
| SDZ^1^ | 39.6 | 90.3 | -71.1 | 5.2 | 34.3 | 105 | -76.7 | 1.1 | 35.3 | 110 | -78.2 | 3.7 | 0.998 |
| AcSDZ | 77.2 | 101 | -37.3 | 5.7 | 78.9 | 75.4 | -43.5 | 4.8 | 73.4 | 95.8 | -45.8 | 2.9 | 0.993 |
| SMZ | 40.7 | 104 | -46.3 | 3.6 | 38.0 | 101 | -38.9 | 0.8 | 46.5 | 97.6 | -45.4 | 2.1 | 0.988 |
| AcSMZ | 96.3 | 102 | -3.01 | 5.4 | 84.8 | 84.8 | -10.4 | 1.9 | 76.1 | 92.0 | -10.7 | 2.6 | 0.991 |

^1^Spike levels: 10, 22.5 and 45 ng g^-1^ dw; ^2^Spike levels: 25, 35 and 45 ng g^-1^ dw; Parent compounds are marked in bold.

**Table S7.** Recovery (R), accuracy (A), matrix effect (ME), precision of the method expressed as relative standard deviation (RSD) and matrix-matched calibration curves correlation coefficients (R^2^) for compost.

| Compound | Low level (1.5 ng g^-1^ dw) | | | | Medium level (22.5 ng g^-1^ dw) | | | | High level (45 ng g^-1^ dw) | | | | R^2^ | |
| --- | --- | --- | --- | --- | --- | --- | --- | --- | --- | --- | --- | --- | --- | --- |
|  | R  (%) | A  (%) | ME  (%) | RSD  (%) | R  (%) | A  (%) | ME  (%) | RSD  (%) | R  (%) | A  (%) | ME  (%) | RSD  (%) |  |  |
| RXM | 82.2 | 88.8 | -77.2 | 15 | 85.1 | 96.2 | -81.9 | 7.2 | 82.8 | 97.5 | -80.6 | 5.7 | 0.992 |  |
| AZM | 92.1 | 96.8 | -26.7 | 29 | 78.1 | 99.6 | -27.4 | 5.1 | 98.1 | 101 | -18.7 | 5.5 | 0.966 |  |
| ERY | 96.0 | 104 | -80.4 | 20 | 71.9 | 91.9 | -74.1 | 12 | 77.4 | 96.9 | -79.3 | 8.6 | 0.991 |  |
| CLM | 64.9 | 111 | -85.5 | 21 | 68.9 | 104 | -72.8 | 4.7 | 68.4 | 102 | -77.6 | 8.8 | 0.984 |  |
| DM-CLM | 90.9 | 106 | -89.6 | 23 | 72.2 | 101 | -80.4 | 6.4 | 81.1 | 95.7 | -84.3 | 8.5 | 0.994 |  |
| NOR | 50.5 | 72.8 | -62.3 | 7.7 | 49.5 | 97.9 | -77.6 | 5.9 | 31.4 | 100 | -70.4 | 1.7 | 0.982 |  |
| ENR | 55.4 | 77.1 | -60.2 | 7.1 | 36.2 | 99.9 | -67.9 | 6.2 | 23.4 | 89.3 | -67.4 | 0.9 | 0.989 |  |
| CIP | 68.9 | 80.0 | -68.8 | 7.2 | 38.0 | 100 | -68.3 | 4.5 | 17.2 | 96.1 | -54.6 | 2.1 | 0.976 |  |
| TC^1^ | 11.4 | 95.8 | -8.61 | 15 | 12.8 | 94.9 | -2.42 | 17 | 25.6 | 97.0 | -1.94 | 19 | 0.989 |  |
| EP-TC^1^ | 11.2 | 68.4 | -6.26 | 12 | 11.5 | 101 | -2.08 | 12 | 11.0 | 93.9 | -1.89 | 7.8 | 0.920 |  |
| TMP | 58.5 | 97.5 | -38.2 | 3.1 | 57.9 | 99.8 | -38.2 | 0.6 | 59.3 | 100 | -35.3 | 3.5 | 0.996 |  |
| 4-OH-TMP^2^ | 60.6 | 98.9 | -2.25 | 6.1 | 70.7 | 96.9 | -12.5 | 5.9 | 68.0 | 96.5 | -8.11 | 4.6 | 0.980 |  |
| DM-TMP | 43.3 | 93.5 | -56.6 | 6.0 | 54.7 | 102 | -51.0 | 1.9 | 44.9 | 102 | -48.5 | 3.0 | 0.998 |  |
| SMX | 46.4 | 94.9 | -25.5 | 7.9 | 58.6 | 96.9 | -9.47 | 6.0 | 33.7 | 101 | -10.4 | 6.3 | 0.990 |  |
| AcSMX | 92.0 | 103 | -14.9 | 18 | 86.9 | 99.9 | -15.5 | 0.9 | 88.5 | 95.9 | -15.0 | 4.5 | 0.992 |  |
| SMX-GL^2^ | 52.3 | 99.9 | -17.8 | 21 | 58.6 | 96.7 | -16.5 | 22 | 57.5 | 96.1 | -11.9 | 7.0 | 0.995 |  |
| SDZ | 44.6 | 108 | -25.4 | 29 | 43.1 | 100 | -23.8 | 11 | 40.6 | 99.9 | -23.5 | 2.9 | 0.998 |  |
| AcSDZ | 85.4 | 101 | -37.3 | 10 | 79.3 | 96.9 | -43.5 | 1.3 | 81.2 | 96.4 | -45.8 | 4.7 | 0.996 |  |
| SMZ | 44.1 | 91.9 | -11.0 | 7.2 | 40.5 | 97.8 | -2.30 | 7.6 | 49.0 | 97.2 | -1.93 | 3.5 | 0.990 |  |
| AcSMZ | 87.0 | 109 | -6.31 | 16 | 84.4 | 98.1 | -6.75 | 1.2 | 85.8 | 98.3 | -4.16 | 2.8 | 0.995 |  |

^1^Spike levels: 25, 35 and 45 ng g^-1^ dw; ^2^Spike levels 10, 22.5 and 45 ng g^-1^ dw. Parent compounds are marked in bold.

**Table S8.** Recovery (R), accuracy (A), matrix effect (ME), precision of the method expressed as relative standard deviation (RSD) and matrix-matched calibration curves correlation coefficients (R^2^) for soil.

| Compound | Low level (1.5 ng g^-1^ dw) | | | | Medium level (22.5 ng g^-1^ dw) | | | | High level (45 ng g^-1^ dw) | | | | R^2^ |
| --- | --- | --- | --- | --- | --- | --- | --- | --- | --- | --- | --- | --- | --- |
|  | R (%) | A (%) | ME (%) | RSD  (%) | R (%) | A (%) | ME (%) | RSD  (%) | R (%) | A  (%) | ME (%) | RSD  (%) |  |
| RXM | 84.2 | 97.9 | -4.83 | 2.3 | 86.1 | 97.9 | -5.45 | 0.7 | 95.8 | 97.3 | -0.07 | 4.7 | 0.997 |
| AZM | 95.2 | 100 | -6.74 | 2.9 | 97.8 | 100 | -0.72 | 2.8 | 102 | 99.8 | -7.59 | 3.7 | 0.991 |
| ERY | 104 | 99.6 | -57.2 | 3.2 | 93.2 | 98.7 | -54.1 | 1.3 | 82.0 | 101 | -36.2 | 0.7 | 0.994 |
| CLM | 89.2 | 101 | -49.3 | 3.2 | 75.2 | 101 | -50.1 | 2.1 | 76.4 | 98.6 | -39.0 | 2.9 | 0.994 |
| DM-CLM | 105 | 102 | -57.2 | 4.6 | 94.0 | 102 | -50.9 | 2.4 | 78.7 | 100 | -37.0 | 1.7 | 0.993 |
| NOR | 92.7 | 99.9 | -40.6 | 1.6 | 94.9 | 104 | -41.1 | 3.5 | 99.0 | 99.0 | -42.8 | 5.2 | 0.994 |
| ENR | 93.7 | 104 | -46.2 | 4.8 | 82.8 | 99.5 | -46.3 | 24 | 52.3 | 97.9 | -58.6 | 2.1 | 0.995 |
| CIP | 54.4 | 95.8 | -35.1 | 0.7 | 47.5 | 96.7 | -34.1 | 1.7 | 43.1 | 99.3 | -36.6 | 2.4 | 0.985 |
| TC^1^ | 15.6 | 69.8 | -4.28 | 13 | 18.4 | 97.8 | -1.47 | 13 | 17.1 | 95.8 | -9.50 | 10 | 0.986 |
| EP-TC^1^ | 50.2 | 99.1 | -1.82 | 8.8 | 58.0 | 95.8 | -1.52 | 11 | 58.1 | 98.1 | -8.76 | 9.8 | 0.985 |
| TMP | 95.2 | 100 | -4.57 | 2.6 | 97.8 | 98.1 | -1.59 | 2.7 | 102 | 99.2 | -0.56 | 2.6 | 0.994 |
| 4-OH-TMP^2^ | 92.1 | 94.3 | -2.11 | 13 | 76.0 | 96.9 | -4.48 | 15 | 62.7 | 99.1 | -1.80 | 4.6 | 0.988 |
| DM-TMP | 54.4 | 99.5 | -5.90 | 2.6 | 59.0 | 95.9 | -0.50 | 1.4 | 64.6 | 98.7 | -3.05 | 2.1 | 0.997 |
| SMX | 69.0 | 101 | -2.26 | 5.9 | 85.1 | 97.3 | -0.74 | 15 | 76.1 | 105 | -1.25 | 10 | 0.992 |
| AcSMX | 91.9 | 96.7 | -2.23 | 3.8 | 91.0 | 98.4 | -0.05 | 1.5 | 92.7 | 103 | -1.35 | 1.1 | 0.992 |
| SMX-GL^2^ | 79.8 | 98.6 | -14.7 | 31 | 96.1 | 99.5 | -8.48 | 3.5 | 82.0 | 102 | -3.51 | 14 | 0.997 |
| SDZ | 79.1 | 97.1 | -0.96 | 4.9 | 86.7 | 97 | -1.54 | 16 | 83.2 | 97.9 | -1.00 | 9.0 | 0.991 |
| AcSDZ | 84.3 | 96.9 | -5.66 | 14 | 98.7 | 96.9 | -2.29 | 1.3 | 93.1 | 101 | -2.22 | 2.2 | 0.994 |
| SMZ | 58.1 | 98.2 | -14.7 | 4.8 | 66.4 | 96.9 | -2.72 | 13 | 68.2 | 99.8 | -1.16 | 6.3 | 0.994 |
| AcSMZ | 93.1 | 97.3 | -1.90 | 1.4 | 100 | 99 | -1.91 | 2.4 | 99.8 | 100 | -2.68 | 0.8 | 0.993 |

^1^Spike levels: 25, 35 and 45 ng g^-1^ dw; ^2^Spike levels: 5, 22.5 and 45 ng g^-1^ dw; Parent compounds are marked in bold.

**Table S9.** Application of the method to five different primary sludge samples spiked at three concentration levels for method robustness assessment. RSD: relative standard deviation.

| Compound | Low level (1.5 ng g^-1^ dw) | | | | Medium level (22.5 ng g^-1^ dw) | | | | High level (45 ng g^-1^ dw) | | | |
| --- | --- | --- | --- | --- | --- | --- | --- | --- | --- | --- | --- | --- |
|  | Found  (ng g^-1^ dw) | Standard  deviation  (ng g^-1^ dw) | *t*_Student_ | RSD  (%) | Found  (ng g^-1^ dw) | Standard  deviation  (ng g^-1^ dw) | *t*_Student_ | RSD  (%) | Found  (ng g^-1^ dw) | Standard  deviation  (ng g^-1^ dw) | *t*_Student_ | RSD  (%) |
| RXM^1^ | 8.1 | 1.5 | -2.83 | 19 | 17.4 | 4.4 | -2.59 | 25 | 37.2 | 6.4 | -2.73 | 17 |
| AZM^1^ | 7.9 | 1.7 | -2.76 | 22 | 17.5 | 4.6 | -2.41 | 26 | 36.8 | 6.7 | -2.74 | 18 |
| ERY^1^ | 7.8 | 1.8 | -2.73 | 23 | 17.7 | 3.8 | -2.85 | 22 | 39.1 | 3.7 | -3.57 | 9 |
| CLM | 1.2 | 0.3 | -2.03 | 28 | 20.6 | 3.1 | -1.38 | 15 | 41.2 | 3.8 | -2.24 | 9 |
| DM-CLM^1^ | 7.2 | 2.1 | -3.05 | 28 | 18.4 | 3.3 | -2.76 | 18 | 39.5 | 4.5 | -2.73 | 11 |
| NOR | 1.6 | 0.4 | 0.64 | 22 | 19.1 | 2.9 | -2.62 | 15 | 36.8 | 6.9 | -2.66 | 19 |
| ENR | 1.4 | 0.3 | -0.89 | 18 | 18.9 | 3.2 | -2.52 | 17 | 36.4 | 7.0 | -2.75 | 19 |
| CIP | 1.5 | 0.3 | 0.00 | 22 | 16.8 | 4.6 | -2.77 | 27 | 37.8 | 5.8 | -2.78 | 15 |
| TC^2^ | 24.3 | 3.4 | -0.46 | 14 | 29.3 | 5.2 | -2.45 | 18 | 38.5 | 7.2 | -2.02 | 19 |
| EP-TC^2^ | 24.5 | 4.2 | -0.27 | 17 | 31.9 | 5.3 | -1.31 | 17 | 36.3 | 7.8 | -2.49 | 21 |
| TMP | 1.4 | 0.3 | -1.08 | 23 | 21.3 | 2.1 | -1.28 | 10 | 39.3 | 6.1 | -2.09 | 16 |
| 4-OH-TMP^1^ | 9.6 | 1.9 | -0.47 | 20 | 21.1 | 2.9 | -1.08 | 14 | 39.1 | 5.3 | -2.49 | 14 |
| DM-TMP | 1.5 | 0.4 | -0.31 | 25 | 20.9 | 1.8 | -1.99 | 9 | 44.8 | 7.3 | -0.06 | 16 |
| SMX | 1.4 | 0.3 | -1.05 | 24 | 17.9 | 3.9 | -2.64 | 22 | 40.2 | 9.7 | -1.11 | 24 |
| AcSMX | 1.5 | 0.4 | -0.25 | 25 | 18.2 | 3.8 | -2.53 | 21 | 38.8 | 5.3 | -2.62 | 14 |
| SMX-GL^1^ | 9.8 | 1.6 | -0.31 | 16 | 20.6 | 4.2 | -1.01 | 20 | 36.2 | 8.8 | -2.24 | 24 |
| SDZ^1^ | 9.6 | 1.7 | -0.58 | 18 | 20.1 | 3.2 | -1.68 | 16 | 41.9 | 3.7 | -1.87 | 9 |
| AcSDZ | 1.5 | 0.3 | -0.39 | 20 | 18.2 | 3.6 | -2.67 | 20 | 38.5 | 5.3 | -2.74 | 14 |
| SMZ | 1.5 | 0.4 | -0.13 | 24 | 20.7 | 3.6 | -1.12 | 17 | 42.4 | 5.3 | -1.10 | 13 |
| AcSMZ | 1.6 | 0.4 | 0.73 | 25 | 18.3 | 3.4 | -2.76 | 19 | 39.3 | 5.6 | -2.28 | 14 |

^1^Spike levels: 10, 22.5 and 45 ng g^-1^ dw; ^2^Spike levels: 25, 35 and 45 ng g^-1^ dw; Parent compounds are marked in bold.

**Table S10.** Application of the method to five different secondary sludge samples spiked at three concentration levels for method robustness assessment. RSD: relative standard deviation.

| Compound | Low level (1.5 ng g^-1^ dw) | | | | Medium level (22.5 ng g^-1^ dw) | | | | High level (45 ng g^-1^ dw) | | | |
| --- | --- | --- | --- | --- | --- | --- | --- | --- | --- | --- | --- | --- |
|  | Found  (ng g^-1^ dw) | Standard  deviation  (ng g^-1^ dw) | *t*_Student_ | RSD  (%) | Found  (ng g^-1^ dw) | Standard  deviation  (ng g^-1^ dw) | *t*_Student_ | RSD  (%) | Found  (ng g^-1^ dw) | Standard  deviation  (ng g^-1^ dw) | *t*_Student_ | RSD  (%) |
| RXM^1^ | 9.9 | 0.8 | -0.42 | 8 | 19.3 | 2.6 | -2.75 | 13 | 48.2 | 5.6 | 1.28 | 12 |
| AZM^1^ | 10.1 | 1.1 | 0.20 | 11 | 19.6 | 2.3 | -2.82 | 12 | 41.3 | 3.8 | -2.18 | 9 |
| ERY^1^ | 9.1 | 1.9 | -1.06 | 21 | 20.6 | 2.8 | -1.52 | 14 | 42.1 | 2.7 | -2.40 | 6 |
| CLM | 1.4 | 0.3 | -0.86 | 19 | 19.5 | 2.5 | -2.68 | 13 | 42.5 | 6.9 | -0.81 | 16 |
| DM-CLM^1^ | 10.3 | 0.9 | 0.74 | 9 | 18.4 | 3.3 | -2.78 | 18 | 41.9 | 5.8 | -1.20 | 14 |
| NOR | 1.4 | 0.3 | -1.29 | 19 | 18.8 | 3.4 | -2.43 | 18 | 39.8 | 4.6 | -2.53 | 12 |
| ENR | 1.6 | 0.4 | 0.32 | 23 | 18.6 | 3.2 | -2.73 | 17 | 42.1 | 5.2 | -1.25 | 12 |
| CIP | 1.4 | 0.3 | -0.54 | 20 | 19.2 | 2.7 | -2.73 | 14 | 41.2 | 3.9 | -2.18 | 9 |
| TC^2^ | 21.8 | 2.6 | -2.75 | 12 | 29.9 | 4.3 | -2.65 | 14 | 40.6 | 3.8 | -2.59 | 9 |
| EP-TC^2^ | 22.1 | 2.8 | -2.32 | 13 | 31.2 | 3.2 | -2.66 | 10 | 40.3 | 3.9 | -2.69 | 10 |
| TMP | 1.8 | 0.3 | 2.40 | 16 | 24.3 | 2.3 | 1.75 | 9 | 43.8 | 4.8 | -0.56 | 11 |
| 4-OH-TMP^1^ | 8.9 | 0.9 | -2.73 | 10 | 21.8 | 1.4 | -1.12 | 6 | 41.2 | 3.4 | -2.50 | 8 |
| DM-TMP | 1.4 | 0.2 | -2.24 | 11 | 19.3 | 2.8 | -2.56 | 15 | 43.2 | 3.9 | -1.03 | 9 |
| SMX | 1.7 | 0.2 | 2.10 | 10 | 21.9 | 1.2 | -1.12 | 5 | 42.1 | 2.8 | -2.32 | 7 |
| AcSMX | 1.7 | 0.2 | 2.45 | 12 | 19.6 | 3.1 | -2.09 | 16 | 39.6 | 4.4 | -2.74 | 11 |
| SMX-GL^1^ | 9.4 | 0.6 | -2.24 | 6 | 20.5 | 2.3 | -1.94 | 11 | 40.8 | 3.9 | -2.41 | 10 |
| SDZ^1^ | 9.9 | 0.9 | -0.25 | 9 | 23.2 | 1.9 | 0.82 | 8 | 47.9 | 3.1 | 2.09 | 6 |
| AcSDZ | 1.6 | 0.2 | 0.89 | 10 | 19.8 | 2.8 | -2.16 | 14 | 39.9 | 4.6 | -2.48 | 12 |
| SMZ | 1.4 | 0.2 | -1.40 | 11 | 23.0 | 1.2 | 0.93 | 5 | 45.7 | 1.9 | 0.82 | 4 |
| AcSMZ | 1.5 | 0.1 | 0.00 | 8 | 18.6 | 3.2 | -2.73 | 17 | 39.3 | 4.6 | -2.77 | 12 |

^1^Spike levels: 10, 22.5 and 45 ng g^-1^ dw; ^2^Spike levels: 25, 35 and 45 ng g^-1^ dw; Parent compounds are marked in bold.

**Table S11.** Application of the method application to five different digested sludge samples spiked at three concentration levels for method robustness assessment. RSD: relative standard deviation.

| Compound | Low level (1.5 ng g^-1^ dw) | | | | Medium level (22.5 ng g^-1^ dw) | | | | High level (45 ng g^-1^ dw) | | | |
| --- | --- | --- | --- | --- | --- | --- | --- | --- | --- | --- | --- | --- |
|  | Found  (ng g^-1^ dw) | Standard  deviation  (ng g^-1^ dw) | *t*_Student_ | RSD  (%) | Found  (ng g^-1^ dw) | Standard  deviation  (ng g^-1^ dw) | *t*_Student_ | RSD  (%) | Found  (ng g^-1^ dw) | Standard  deviation  (ng g^-1^ dw) | *t*_Student_ | RSD  (%) |
| RXM^1^ | 9.9 | 0.5 | -0.44 | 5 | 21.8 | 0.8 | -1.91 | 4 | 42.1 | 3.9 | -1.66 | 9 |
| AZM^1^ | 10.0 | 0.4 | -0.27 | 4 | 21.3 | 3.6 | -0.75 | 17 | 41.2 | 3.1 | -2.74 | 8 |
| ERY^1^ | 10.2 | 0.7 | 0.63 | 7 | 19.4 | 2.8 | -2.48 | 14 | 37.3 | 6.2 | -2.78 | 17 |
| CLM | 1.6 | 0.2 | 1.94 | 9 | 23.0 | 1.6 | 0.70 | 7 | 45.1 | 1.5 | 0.15 | 3 |
| DM-CLM^1^ | 9.5 | 0.9 | -1.23 | 10 | 22.1 | 1.7 | -0.53 | 8 | 40.9 | 3.4 | -2.70 | 8 |
| NOR | 1.3 | 0.2 | -2.43 | 18 | 22.0 | 2.9 | -0.39 | 13 | 41.2 | 3.6 | -2.36 | 9 |
| ENR | 1.5 | 0.1 | -1.24 | 6 | 19.8 | 2.2 | -2.74 | 11 | 40.2 | 3.9 | -2.75 | 10 |
| CIP | 1.5 | 0.1 | -1.12 | 5 | 20.1 | 2.0 | -2.68 | 10 | 40.3 | 3.8 | -2.77 | 9 |
| TC^2^ | 22.2 | 2.3 | -2.69 | 10 | 33.2 | 3.3 | -1.22 | 10 | 43.0 | 3.9 | -1.15 | 9 |
| EP-TC^2^ | 25.3 | 2.5 | 0.26 | 10 | 31.8 | 3.1 | -2.31 | 10 | 40.5 | 3.7 | -2.72 | 9 |
| TMP | 1.8 | 0.3 | 2.68 | 14 | 23.5 | 0.9 | 2.48 | 4 | 43.1 | 2.1 | -2.02 | 5 |
| 4-OH-TMP^1^ | 10.9 | 0.7 | 2.76 | 7 | 21.2 | 1.1 | -2.64 | 5 | 44.2 | 1.1 | -1.63 | 2 |
| DM-TMP | 1.3 | 0.2 | -2.63 | 13 | 21.7 | 0.9 | -1.99 | 4 | 45.6 | 1.9 | 0.71 | 4 |
| SMX | 1.3 | 0.2 | -2.66 | 17 | 21.3 | 1.5 | -1.79 | 7 | 43.2 | 1.8 | -2.24 | 4 |
| AcSMX | 1.7 | 0.2 | 1.68 | 12 | 23.1 | 0.9 | 1.49 | 4 | 46.2 | 1.9 | 1.41 | 4 |
| SMX-GL^1^ | 9.8 | 0.8 | -0.56 | 8 | 20.8 | 1.5 | -2.53 | 7 | 45.1 | 5.6 | 0.04 | 12 |
| SDZ^1^ | 9.3 | 0.6 | -2.61 | 6 | 24.3 | 1.7 | 2.37 | 7 | 45.3 | 2.7 | 0.25 | 6 |
| AcSDZ | 1.5 | 0.1 | 0.00 | 7 | 21.1 | 1.6 | -1.96 | 8 | 43.5 | 1.6 | -2.10 | 4 |
| SMZ | 1.6 | 0.1 | 2.73 | 6 | 23.0 | 0.7 | 1.60 | 3 | 43.1 | 2.1 | -2.02 | 5 |
| AcSMZ | 1.5 | 0.1 | -0.41 | 7 | 21.5 | 0.9 | -2.48 | 4 | 41.8 | 2.6 | -2.75 | 6 |

^1^Spike levels: 10, 22.5 and 45 ng g^-1^ dw; ^2^Spike levels: 25, 35 and 45 ng g^-1^ dw; Parent compounds are marked in bold.

**Table S12.** Application of the method to five different compost sludge samples spiked at three concentration levels for method robustness assessment. RSD: relative standard deviation.

| Compound | Low level (1.5 ng g^-1^ dw) | | | | Medium level (22.5 ng g^-1^ dw) | | | | High level (45 ng g^-1^ dw) | | | |
| --- | --- | --- | --- | --- | --- | --- | --- | --- | --- | --- | --- | --- |
|  | Found  (ng g^-1^ dw) | Standard  deviation  (ng g^-1^ dw) | *t*_Student_ | RSD  (%) | Found  (ng g^-1^ dw) | Standard  deviation  (ng g^-1^ dw) | *t*_Student_ | RSD  (%) | Found  (ng g^-1^ dw) | Standard  deviation  (ng g^-1^ dw) | *t*_Student_ | RSD  (%) |
| RXM | 1.3 | 0.2 | -2.24 | 17 | 21.5 | 1.6 | -1.38 | 8 | 43.5 | 2.5 | -1.33 | 8 |
| AZM | 1.4 | 0.2 | -0.85 | 15 | 22.5 | 1.3 | 0.00 | 6 | 45.2 | 0.5 | 0.93 | 6 |
| ERY | 1.6 | 0.3 | 0.77 | 20 | 20.8 | 2.6 | -1.46 | 13 | 43.5 | 3.8 | -0.88 | 13 |
| CLM | 1.6 | 0.3 | 0.94 | 19 | 23.8 | 1.5 | 1.94 | 6 | 45.1 | 4.2 | 0.05 | 6 |
| DM-CLM | 1.6 | 0.3 | 0.69 | 18 | 22.5 | 1.6 | 0.00 | 7 | 43.7 | 3.9 | -0.75 | 7 |
| NOR | 1.3 | 0.2 | -2.61 | 14 | 22.3 | 1.8 | -0.25 | 8 | 45.2 | 1.0 | 0.47 | 8 |
| ENR | 1.3 | 0.2 | -2.02 | 16 | 22.1 | 1.8 | -0.51 | 8 | 41.8 | 2.6 | -2.75 | 8 |
| CIP | 1.3 | 0.2 | -2.53 | 11 | 22.4 | 1.5 | -0.15 | 6 | 43.1 | 2.1 | -2.02 | 6 |
| TC^1^ | 24.3 | 3.9 | -0.40 | 16 | 33.8 | 5.7 | -0.47 | 17 | 42.9 | 6.3 | -0.75 | 17 |
| EP-TC^1^ | 21.2 | 3.1 | -2.74 | 15 | 35.1 | 4.4 | 0.05 | 13 | 41.9 | 3.5 | -1.98 | 13 |
| TMP | 1.5 | 0.2 | -0.53 | 14 | 22.9 | 0.5 | 1.79 | 2 | 44.8 | 2.3 | -0.19 | 2 |
| 4-OH-TMP^2^ | 9.9 | 0.8 | -0.25 | 8 | 22.1 | 1.5 | -0.60 | 7 | 43.7 | 2.5 | -1.16 | 7 |
| DM-TMP | 1.3 | 0.2 | -2.24 | 15 | 23.0 | 0.6 | 1.86 | 3 | 45.3 | 1.4 | 0.48 | 3 |
| SMX | 1.4 | 0.3 | -0.67 | 21 | 21.6 | 1.6 | -1.26 | 7 | 45.2 | 2.9 | 0.15 | 7 |
| AcSMX | 1.5 | 0.3 | 0.14 | 20 | 22.1 | 0.8 | -1.12 | 4 | 43.8 | 2.1 | -1.28 | 4 |
| SMX-GL^2^ | 9.9 | 2.2 | -0.15 | 22 | 21.4 | 4.8 | -0.51 | 22 | 43.6 | 3.1 | -1.01 | 22 |
| SDZ | 1.5 | 0.3 | -0.29 | 21 | 22.1 | 2.6 | -0.34 | 12 | 44.9 | 1.4 | -0.16 | 12 |
| AcSDZ | 1.5 | 0.3 | -0.37 | 21 | 22.2 | 0.4 | -1.64 | 2 | 44.1 | 2.2 | -0.94 | 2 |
| SMZ | 1.4 | 0.3 | -0.93 | 21 | 21.9 | 1.7 | -0.78 | 8 | 44.0 | 1.8 | -1.28 | 8 |
| AcSMZ | 1.6 | 0.3 | 0.43 | 20 | 22.3 | 0.5 | -0.86 | 2 | 44.6 | 1.4 | -0.65 | 2 |

^1^Spike levels: 25, 35 and 45 ng g^-1^ dw; ^2^Spike levels: 10, 22.5 and 45 ng g^-1^ dw; Parent compounds are marked in bold.

**Table S13.** Application of the method to five different soil samples spiked at three concentration levels for method robustness assessment. RSD: relative standard deviation.

| Compound | Low level (1.5 ng g^-1^ dw) | | | | Medium level (22.5 ng g^-1^ dw) | | | | High level (45 ng g^-1^ dw) | | | |
| --- | --- | --- | --- | --- | --- | --- | --- | --- | --- | --- | --- | --- |
|  | Found  (ng g^-1^ dw) | Standard  deviation  (ng g^-1^ dw) | *t*_Student_ | RSD  (%) | Found  (ng g^-1^ dw) | Standard  deviation  (ng g^-1^ dw) | *t*_Student_ | RSD  (%) | Found  (ng g^-1^ dw) | Standard  deviation  (ng g^-1^ dw) | *t*_Student_ | RSD  (%) |
| RXM | 1.4 | 0.2 | -1.17 | 15 | 22.2 | 2.1 | -0.32 | 9 | 43.9 | 3.1 | -0.79 | 7 |
| AZM | 1.5 | 0.1 | 0.28 | 5 | 22.3 | 0.8 | -0.53 | 4 | 44.5 | 0.9 | -1.26 | 2 |
| ERY | 1.5 | 0.1 | -0.75 | 6 | 22.2 | 0.4 | -1.69 | 2 | 45.1 | 0.7 | 0.33 | 1 |
| CLM | 1.5 | 0.1 | 0.20 | 7 | 22.9 | 0.6 | 1.40 | 2 | 44.9 | 1.7 | -0.14 | 4 |
| DM-CLM | 1.6 | 0.2 | 0.59 | 12 | 23.2 | 0.7 | 2.20 | 3 | 46.1 | 1.1 | 2.20 | 2 |
| NOR | 1.5 | 0.2 | -0.24 | 13 | 23.6 | 0.9 | 2.67 | 4 | 44.9 | 2.9 | -0.08 | 6 |
| ENR | 1.5 | 0.2 | 0.29 | 15 | 22.8 | 2.3 | 0.29 | 10 | 45.0 | 4.1 | 0.00 | 9 |
| CIP | 1.5 | 0.2 | -0.59 | 13 | 22.9 | 0.6 | 1.47 | 3 | 44.3 | 1.1 | -1.40 | 3 |
| TC^1^ | 22.2 | 2.9 | -2.16 | 13 | 34.9 | 4.3 | -0.05 | 12 | 44.0 | 4.6 | -0.49 | 10 |
| EP-TC^1^ | 24.6 | 3.1 | -0.29 | 13 | 33.8 | 4.2 | -0.64 | 12 | 44.4 | 4.5 | -0.30 | 10 |
| TMP | 1.5 | 0.1 | 0.56 | 5 | 22.3 | 0.9 | -0.50 | 4 | 44.0 | 1.2 | -1.82 | 3 |
| 4-OH-TMP^2^ | 4.8 | 0.7 | -0.68 | 15 | 21.9 | 3.2 | -0.42 | 15 | 43.2 | 2.6 | -1.55 | 6 |
| DM-TMP | 1.4 | 0.1 | -1.57 | 7 | 22.1 | 0.5 | -1.75 | 2 | 44.1 | 1.1 | -1.80 | 3 |
| SMX | 1.5 | 0.1 | 0.56 | 8 | 22.3 | 3.3 | -0.14 | 15 | 46.9 | 4.9 | 0.87 | 10 |
| AcSMX | 1.5 | 0.2 | -0.32 | 14 | 22.4 | 0.4 | -0.55 | 2 | 46.0 | 1.1 | 2.03 | 2 |
| SMX-GL^2^ | 4.9 | 1.1 | -0.22 | 22 | 22.9 | 3.2 | 0.28 | 14 | 45.1 | 4.6 | 0.05 | 10 |
| SDZ | 9.8 | 0.7 | -0.57 | 7 | 22.6 | 2.8 | 0.08 | 12 | 44.7 | 4.2 | -0.16 | 9 |
| AcSDZ | 1.4 | 0.3 | -0.52 | 21 | 22.3 | 0.5 | -0.89 | 2 | 45.0 | 1.2 | 0.00 | 3 |
| SMZ | 1.5 | 0.2 | -0.12 | 12 | 21.9 | 3.1 | -0.43 | 14 | 44.1 | 3.1 | -0.65 | 7 |
| AcSMZ | 1.5 | 0.2 | 0.12 | 13 | 22.5 | 0.7 | 0.00 | 3 | 43.9 | 1.1 | -2.24 | 3 |

^1^Spike levels: 25, 35 and 45 ng g^-1^ dw; ^2^Spike levels: 5, 22.5 and 45 ng g^-1^ dw; Parent compounds are marked in bold.

**Table S14.** Method application to primary sludge (P), secondary sludge (SS), digested sludge (D), compost (C) and soil (S).

| Compound | Concentration (ng g^-1^) dw | | | | | | | | | | | | |
| --- | --- | --- | --- | --- | --- | --- | --- | --- | --- | --- | --- | --- | --- |
|  | Primary sludge | | | Secondary sludge | | | Digested sludge | | | Compost | Soil |  |  |
|  | P1 | P2 | P3 | SS1 | SS2 | SS3 | D1 | D2 | D3 | C1 | S1 | S2 | S3 |
| RXM | 121 | 185 | 193 | 164 | 166 | 158 | 96.9 | 28.7 | 14.6 | 2.41 | 49.2 | 43.6 | 24.6 |
| AZM | 136 | 167 | 189 | 131 | 123 | 185 | 34.6 | 12.1 | 34.6 | - | - | - | - |
| ERY | 119 | 8.22 | - | 18.1 | - | 8.04 | - | 90.2 | - | 10.1 | 18.3 | 12.6 | 18.5 |
| CLM | 167 | 131 | 155 | 13.2 | 182 | 159 | 46.9 | 14.1 | 17.4 | - | 20.6 | 23.6 | 18.3 |
| DM-CLM | 153 | 46.5 | 61.1 | 18.4 | 119 | 99.8 | 3.43 | 12.6 | 31.4 | - | 92.9 | 29.3 | 22.1 |
| NOR | 181 | - | 100 | 13.7 | 112 | 120 | 62.5 | 12.2 | 21.6 | 27.0 | 22.8 | 25.3 | 26.3 |
| ENR | 32.8 | 11.6 | 140 | 24.8 | 118 | 163 | 4.03 | 53.8 | - | 20.8 | 22.6 | 23.6 | 27.4 |
| CIP | 176 | 199 | 105 | 12.8 | 129 | 291 | 11.9 | 96.2 | - | 1.49 | 15.5 | 24.6 | 25.3 |
| TC | - | - | - | - | - | - | - | - | - | - | - | - | - |
| EP-TC | - | - | - | - | - | - | - | - | - | - | - | - | - |
| TMP | 36.7 | 2.28 | 34.7 | 22.8 | 21.5 | 7.66 | 0.54 | 8.60 | 5.67 | - | - | - | - |
| 4-OH-TMP | 74.0 | 8.16 | 33.1 | 72.1 | 3.38 | 3.13 | 16.9 | 2.56 | 14.6 | - | - | - | - |
| DM-TMP | 4.71 | 0.69 | 6.33 | 23.5 | 4.78 | 0.74 | 0.55 | 3.12 | 0.59 | 0.17 | 0.24 | - | - |
| SMX | 37.6 | 30.7 | 27.1 | 91.0 | 49.8 | 6.60 | 11.8 | 38.7 | 21.6 | - | - | - | - |
| AcSMX | 0.27 | - | - | - | 7.42 | - | - | 14.6 | - | - | - | - | - |
| SMX-GL | - | - | - | - | - | - | 7.58 | - | - | - | - | - | - |
| SDZ | 7.37 | 64.9 | 9.37 | 16.7 | 12.7 | 8.24 | 7.63 | 8.99 | 9.26 | - | - | - | - |
| AcSDZ | - | - | - | - | - | - | - | - | - | - | - | - | - |
| SMZ | 78.1 | 57.6 | 55.8 | 81.8 | 56.7 | 0.06 | 18.8 | 0.05 | 29.1 | - | - | - | - |
| AcSMZ | 5.64 | - | - | - | 10.5 | - | 5.65 | 6.48 | 5.95 | 0.53 | - | - | - |
| ∑Antibiotics | 1330 | 913 | 1110 | 704 | 1116 | 1210 | 330 | 403 | 206 | 63 | 242 | 183 | 163 |

-: lower than MDL

**
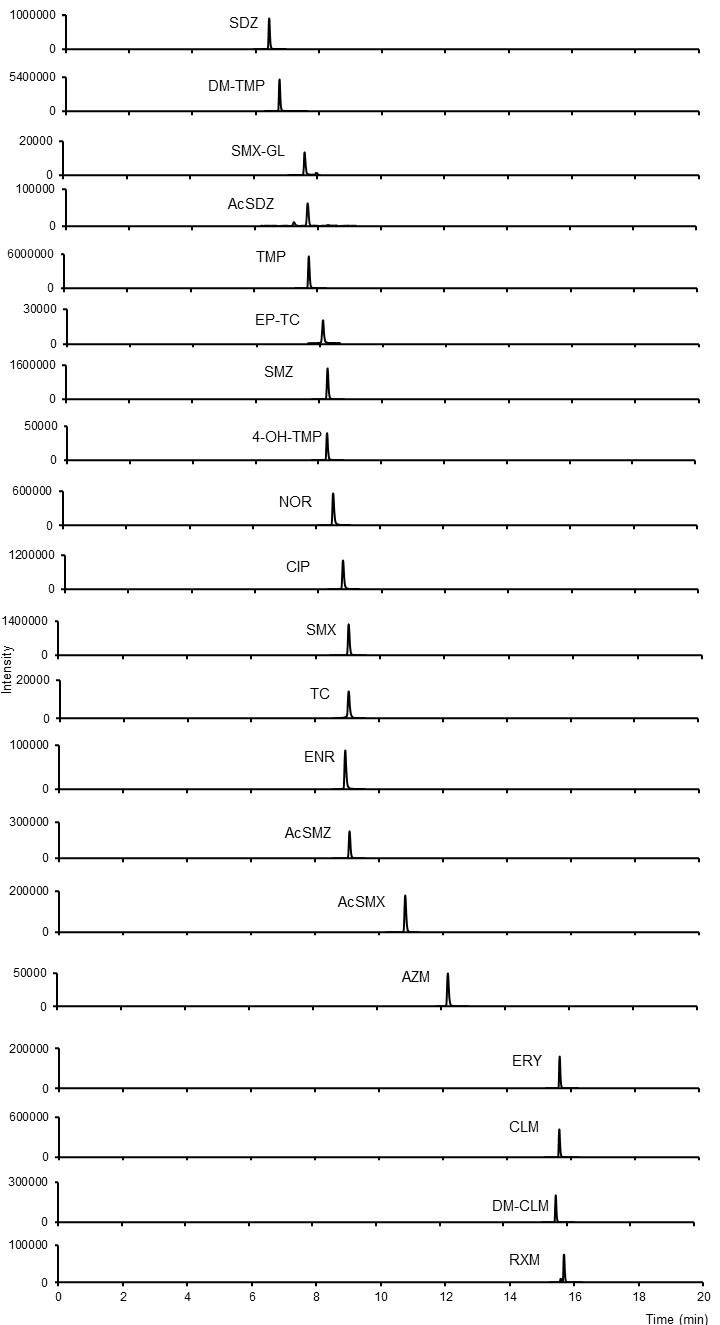
**

**Figure S1.** MRM chromatograms of a primary sludge sample spiked at 35 ng g^-1^ dw (each compound).


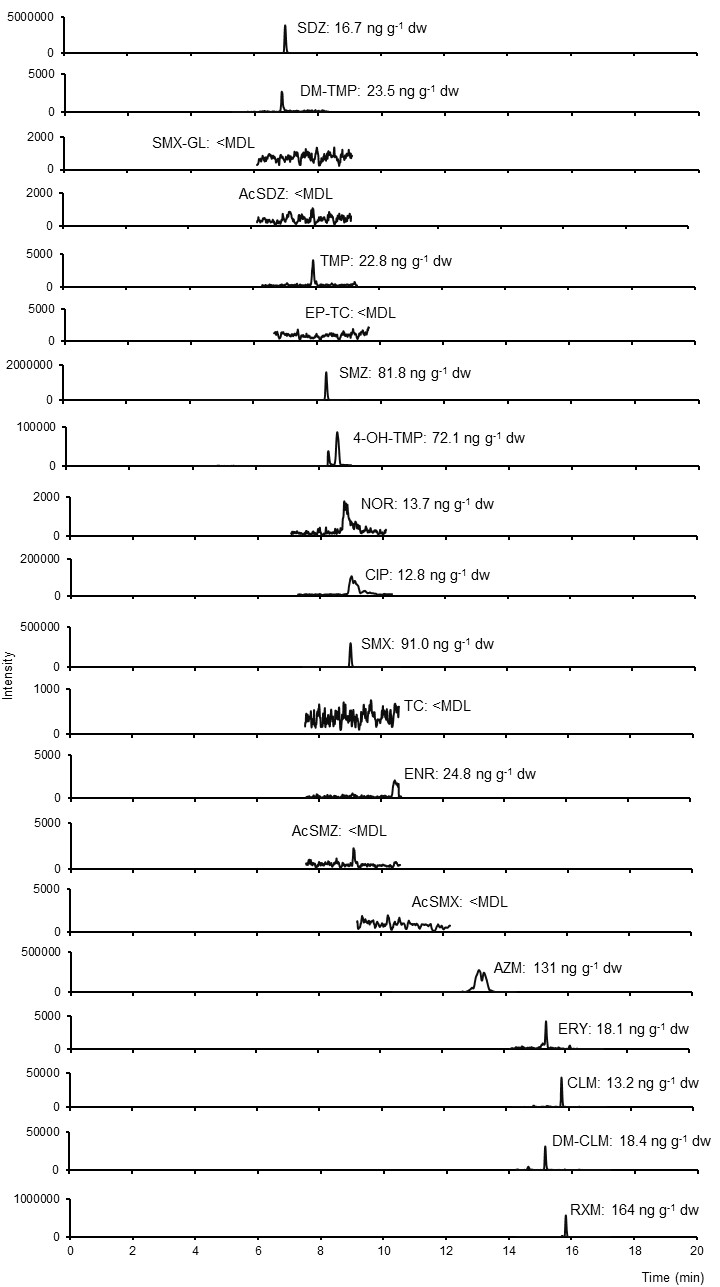


**Figure S2.** MRM chromatograms of a non-spiked secondary sludge sample (sample SS1).
